# An Updated Framework and Signal-to-Noise Analysis of Soil Mass Balance Approaches for Quantifying Enhanced Weathering on Managed Lands

**DOI:** 10.1021/acs.est.5c08303

**Published:** 2025-11-20

**Authors:** Tim Jesper Suhrhoff, Tom Reershemius, Jacob S. Jordan, Shihan Li, Shuang Zhang, Ella Milliken, Boriana Kalderon-Asael, Yael Ebert, Rufaro Nyateka, Jake T. Thompson, Christopher T. Reinhard, Noah J. Planavsky

**Affiliations:** † Yale Center for Natural Carbon Capture, 5755 Yale University, New Haven, Connecticut 06511, United States; ‡ Department of Earth and Planetary Sciences, Yale University, New Haven, Connecticut 06511, United States; § School of Natural and Environmental Sciences, 98458 Newcastle University, Newcastle upon Tyne, England NE1 7RU, United Kingdom; ∥ Mati Carbon, Houston, Texas 77019, United States; ⊥ Department of Oceanography, 2655 Texas A&M University, College Station, Texas 77843, United States; # School of Earth & Atmospheric Sciences, 1372 Georgia Institute of Technology, Atlanta, Georgia 30332, United States

**Keywords:** carbon dioxide removal, enhanced weathering, rock powder dissolution, measurement, reporting, and verification, signal−to−noise analysis, soil mass balance, in−field soil heterogeneity

## Abstract

Enhanced weathering is a promising approach for removing carbon dioxide from the atmosphere at scale while improving agricultural yields. However, accurately quantifying carbon dioxide removal in the field is critical for this approach to scale, particularly given that nearly all the current deployment activity caters to the voluntary carbon market. Here, we present an updated framework and a signal-to-noise analysis for using soil-based mass balance approaches to quantify rock powder dissolution from field-scale data of soil composition. With additional assumptions, the quantification of rock powder dissolution can be used to estimate the carbon dioxide removal potential of EW deployments. The framework we present explicitly accounts for the enrichment of immobile elements in topsoil due to feedstock mass loss, given that omission of this process systematically overestimates feedstock dissolution. We propose and provide support for the idea that feedstock dissolution should be quantified using the sample population mean rather than individual samples. Given the potential for signal-to-noise issues with this framework, it is critical that it is utilized when signals are statistically robust. We present a signal-to-noise analysis based on a new data set of soil cation heterogeneity from high-density spatial sampling of 5 fields (0.6–19.2 samples ha^–1^, 7.1–39.6 pooled cores ha^–1^). The analysis is based on simulated geolocated sample pairs and suggests that detecting rock powder dissolution via soil mass balance should be feasible when application rates, dissolution fractions, and sampling frequencies are above certain threshold values. When planning deployments, signal emergence can be optimized through careful selection of feedstock composition, strategic feedstock application, and improved sampling protocols. Given the potential for signal-to-noise issues within EW projects, protocols cannot exclude fields within projects based on emergence of geochemical signals.

## Graphical abstract

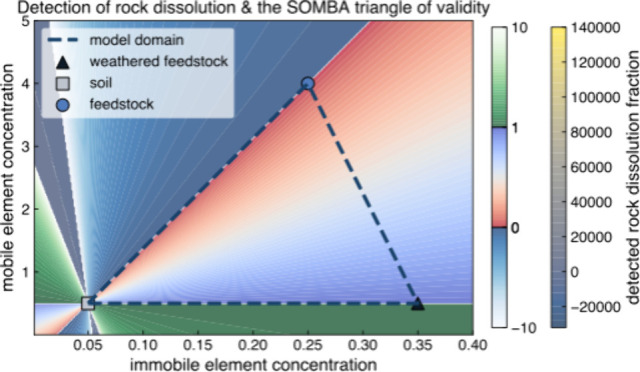

## 1 Introduction

Achieving the climate targets set out by the Paris agreement requires both deep and immediate emissions cuts as well as the ability to remove emitted carbon from the atmosphere.^1–3^ Enhanced Weathering (EW) is one promising approach where CO_2_ can be removed from the atmosphere through the reaction with crushed rock feedstocks applied as soil amendments.^4–12^ In the ideal case, CO_2_ is transferred into bicarbonate and ultimately stored in the oceans for >10 kyrs^13^ or stored as carbonate in both soils and deep-sea sediments. This approach has a unique set of advantages including that carbon is stored more durably compared to many biomass-based approaches. Enhanced weathering can also boost crop yields and does not compete for land resources,^12,14–16^ and the logistics and infrastructure to scale are readily available.

Currently, most CDR activity—including EW—is occurring on the voluntary carbon market.^3,17,18^ This means that CDR credits are primarily being used by companies with net-zero goals to balance ongoing emissions. There is a long tradition of tracking carbon removal in soils through biogeochemical modeling—foremost with soil organic carbon^19^—and using models for emissions offsetting claims.^20,21^ There are also geochemical models for enhanced weathering,^22–25^ and many market actors are keen to explore the use of such models for crediting CDR from EW.^26–28^ However, it has been commonly argued that soil biogeochemical models have not progressed or been sufficiently validated to make them fit for offsetting purposes at this stage.^29,30^ Their limited predictive power mainly stems from large uncertainties in measurements regarding weathering rates and their evolution.^31,32^ Therefore, there is a need to continue to refine a suite of tools to track weathering rates at the field scale.

Tracking carbon fluxes during weathering is a challenge for EW because it is an open-system CDR pathway. However, multiple approaches have been suggested to quantify CDR at the field scale.^33,34^ Broadly speaking, measurement, reporting, and verification (MRV) approaches for EW rely on either solid soil, water, gas, or exchangeable phase measurements.^33^ Soil-based MRV approaches have a unique set of advantages, namely that they yield a time-integrated signal,^33,35^ meaning that they resolve all rock feedstock weathering that occurred between different sampling steps without need for high temporal sampling frequencies.

One promising variation of soil-based MRV approaches is the use of soil mass balance.^33,35,36^ Soil mass balance approaches—here called “SOMBA”—rely on a sample-resample approach where the dissolution of rock powder feedstock is tied to the loss of cations from mixed soil-feedstock samples. The loss of cations provides an estimate of feedstock dissolution, and with additional assumptions can be translated into an estimate of initial CDR. Here, we present an updated framework for this approach that explicitly considers the impact of immobile element enrichment in soils due to feedstock mass loss. Furthermore, we demonstrate some of the intricacies of this approach, perform a signal-to-noise analysis, and share tools to help users constrain rock powder dissolution in their own field deployments. The signal-to-noise analysis is grounded in a new dataset (5 fields, 998 total samples) where spatial heterogeneity in soil major and trace elemental concentration is assessed at a high spatial sampling density.

## 2 Methods

### 2.1 Soil Mass Balance Framework

Soil mass balance approaches leverage that mobile base cations are lost from the solid phase of the soil-feedstock mixture during feedstock weathering, while immobile elements are retained. While base cations mobilized during feedstock weathering may be temporarily retained on the soil exchange complex,^29,31,37,38^ the impact of this process can also be readily quantified or accounted for during sample processing. Using this framework, practitioners can calculate weathering rates of feedstock material based on the mobility of base cations relative to immobile elements.^39–51^ In the context of EW, this framework was first applied in 2023 ^35,52^ and has since been built upon in several publications^12,33,36,53,54^ and preprints.^55–57^

For this approach to be effective, rock feedstock added to fields must be enriched in base cations compared to background soil. If immobile element abundance is also being used to evaluate the amount of feedstock, at least one immobile element needs to be enriched. If these conditions are met, the enrichment of immobile elements in topsoils can be used to constrain rock powder addition, and the loss of cations can be used to estimate rock powder dissolution.^33,35,36^ Using an immobile element to constrain rock powder addition has the benefit that rock powder loss through, e.g., erosion, is not erroneously detected as weathering. However, using a variation of this framework that utilizes geolocated soil sampling after rock application (postspreading) and at a later date (postweathering) could increase the likelihood of generating representative paired data that successfully overcome signal-to-noise limitations. Because first applications of this approach to EW have used Ti as the proxy for rock powder addition, this approach has also been called “TiCAT”,^35^ but because other immobile elements may be used,^36^ we here refer to this approach more broadly as SOMBA.

The loss of cations from topsoils upon weathering can be used to constrain the fraction of rock powder that has dissolved. This in turn can be a proxy for CDR potential, but translating rock powder dissolution into downstream CDR estimates requires additional assumptions as well as quantification of downstream loss processes.^33,35^ These are discussed in detail elsewhere^33,58–60^ and are beyond the scope of this study. Our focus here is to present an updated framework for the quantification of rock powder dissolution, as well as a signal-to-noise analysis of the utility of this approach against background soil heterogeneity. We also share the accompanying code to provide future ERW deployments with a solid foundation for the quantification of rock powder dissolution.

### 2.2 Calculation of Feedstock Dissolution Fraction

#### 2.2.1 Soil Mass Balance and Immobile Element Enrichment due to Mass Loss

After rock powder that has an elevated base cation content ([*j*], with the square brackets denoting concentrations per mass of feedstock) and is enriched in at least one immobile element ([*i*]) is added to fields, the composition of the initial soil-feedstock mixture falls onto a mixing line between the soil and feedstock endmembers (Figure 1a). As the rock powder dissolves, mobile base cations are leached from the mineral phase. This loss of cations is used to quantify the fraction of rock powder that has weathered. This estimate of base cation loss reflects the dissolution of primary feedstock when a chemical extraction of secondary phases or exchangeable cations is performed prior to analysis. Alternatively, the estimate can reflect the proportion of the overall feedstock base cation inventory that has been leached from topsoils entirely if bulk samples are used.

**Figure 1:**
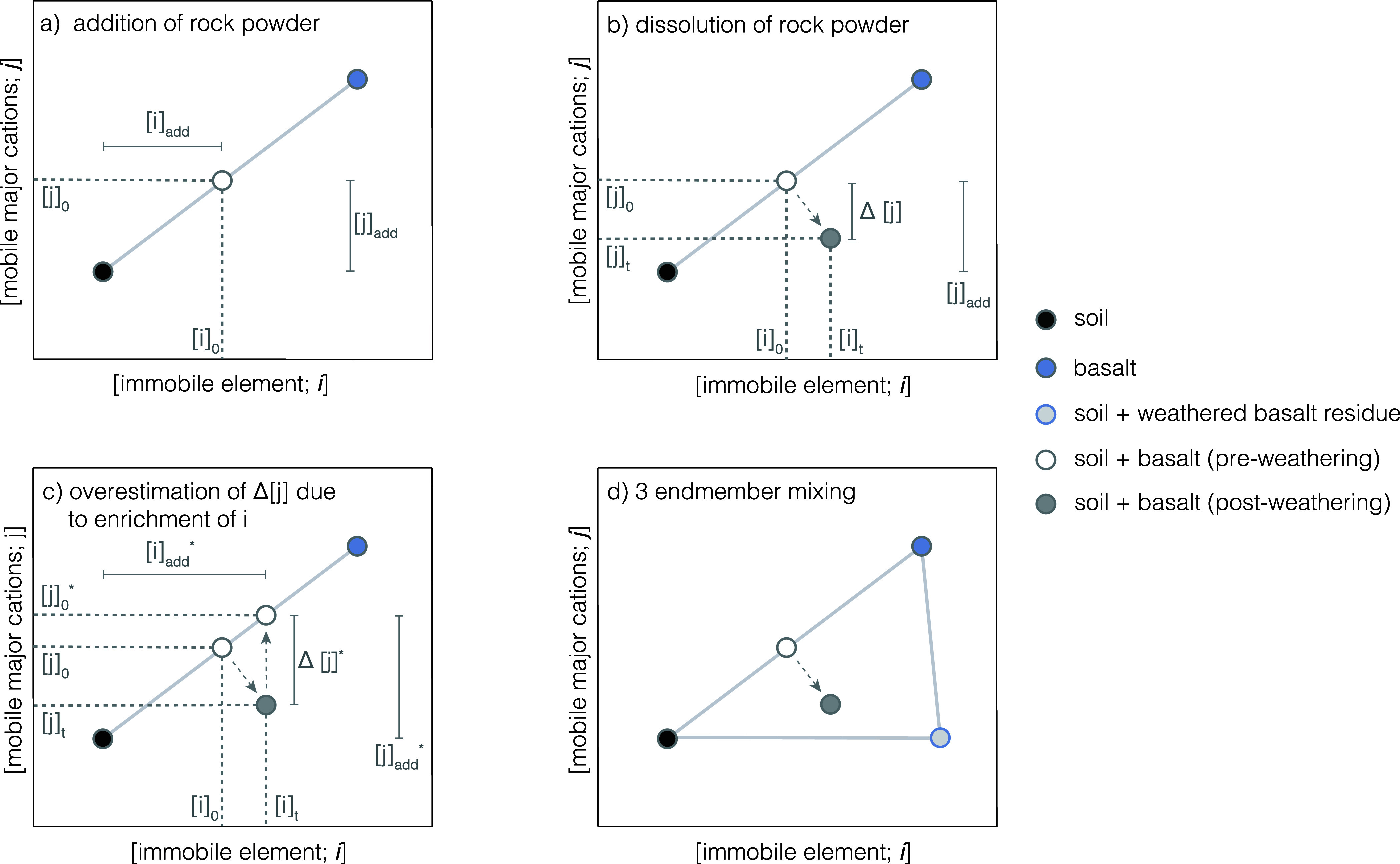
Sketch of the soil-based mass balance framework to quantify rock powder dissolution in soils. After rock powder of an elevated base cation and immobile element concentration compared to baseline soils is added to a field, the composition of the initial soil-feedstock mix falls on the mixing line between both endmembers (a). As feedstock dissolves base cations are released and either stored on the soil exchange complex or flushed out of topsoils. At the same time immobile element concentrations increase as a result of feedstock mass and volume loss, resulting in a vector starting at the preweathering soil-feedstock mix composition toward the bottom right (b). This is important to take into account, because simply projecting the postweathering soil-feedstock mix composition from its immobile element concentration up to the mixing line between soil and feedstock endmembers will cause inflated estimates of cation mass loss and deduced dissolution fractions (c). One way to estimate the dissolution fraction while taking into account the impact of feedstock mass loss is to use a three endmember mixing model where the postweathering composition is described as a mix of the baseline soil, pure feedstock, and a hypothetical weathered feedstock residue endmember (d). Panel d shows the geometry of the three endmember model domain for the simplified case; see section S1.1.3 for a comparison with the nonsimplified case. Note that the offset in immobile element concentrations (i.e., enrichment of immobile element concentrations due to mass loss) for the postweathering soil-feedstock mix sample is exaggerated in panels b–d for the purpose of visualization. In a realistic system the horizontal component of this vector would be smaller compared to the vector between basalt as well as soil + weathered basalt residue (proportionally to the position of the preweathering soil-feedstock mix composition on the missing line). Furthermore, the location of pre- and postweathering soil-basalt mixtures in the figures above is chosen for illustrative purposes and is usually closer to the soil endmember (see also Figure S14).

Enrichment of immobile elements through rock powder dissolution occurs when these are retained in topsoil while a soluble fraction of feedstock is lost from the system. It is typically assumed that the lost feedstock in a sample is replaced with soil that also contains immobile elements in addition to the retained immobile elements added via the rock powder. Furthermore, if the density of feedstock is greater than that of soil, as is true for most cases, this means that the mass being used to calculate the concentration [*i*] is less than for the initial soil-feedstock mixture, such that [*i*]_*t=n*_ > [*i*]_*t=*0_ in all cases where feedstock is partially dissolved. As a result of cation loss and immobile element enrichment, the soil-feedstock mixture composition evolves from the preweathering composition on the mixing line along a vector toward the bottom right in [*j*] vs [*i*] space (Figure 1b).

#### 2.2.2 Quantification of Rock Powder Dissolution

One way to calculate the dissolution fraction (here denoted as the mass transfer coefficient τ_*j*_, used synonymously to dissolution fraction in this manuscript) is from the loss of cations compared to the preweathering soil-feedstock mix

1 $$\tau_{j}=\frac{\Delta \left[j\right]}{{\left[j\right]}_{add}}=\frac{{\left[j\right]}_{t=0}-{\left[j\right]}_{t=n}}{{\left[j\right]}_{add}}$$

2 $${\left[j\right]}_{add}={\left[j\right]}_{0}-{\left[j\right]}_{s}$$

where [*j*]_add_ is the increase in base cation concentrations due to the addition of rock powder, Δ­[*j*] reflects the decrease of base cation concentrations due to feedstock dissolution (calculated as [*j*]_*t*=0_ – [*j*]_*t*=*n*_, i.e., a positive value provided cations are lost), and the subscript *s* corresponds to baseline soil. If the effect of immobile element enrichment is not taken into account, and the fraction of feedstock in the preweathering soil-feedstock mix and associated cation addition is calculated simply by vertically projecting the postweathering composition onto the mixing line (Figure 1c), the estimate of the cation loss from topsoils (Δ­[*j*]*) is inflated, such that the erroneous estimate *τ*_*j*_ * would be larger than *τ*_*j*_. The impact of this enrichment process on postweathering soil concentrations as well as estimates of the fraction of feedstock that has dissolved is discussed in section 3.1.

An alternative way to calculate the fraction of rock powder that has dissolved without exact knowledge of the preweathering soil-feedstock mix composition is to describe the postweathering composition as a mix of three endmembers: pure soil, pure feedstock, as well as the composition of a hypothetical weathered feedstock residue endmember (Figure 1d). The composition of this hypothetical endmember is defined to be the composition that a layer of soil would have after a layer of pure feedstock (corresponding to the soil sampling depth, *d*_sample_) has dissolved. Assuming mass and volume conservation, this endmember mixing approach can be described such that each endmember contributes a volume proportion (*X*) to the observed postweathering composition, which together sum to unity

3 $$X_{s}+X_{f}+X_{wf}=1$$

where subscripts s, f, and wf correspond to baseline soil, feedstock, and weathered feedstock. Because in practical field sampling based on constant soil sampling depths, a system of constant *volume* is sampled, these endmember contributions reflect *volume* contributions to the sampled soil volume defined by the sampling depth over a given area (all calculations and code shared here use 1 hectare (ha) by default).

The contributions of the weathered and remaining feedstock change through time as rock powder dissolves, such that their relative proportions can be used to quantify the fraction of rock powder that has dissolved (see S1 for full derivation):

4 $$\tau_{j}=\frac{X_{wf}}{X_{wf}+X_{f}}$$

A key advantage of this version of the SOMBA framework is that it does not require an immediate postapplication sample; dissolution can be constrained from baseline and postweathering data alone. However, if postapplication sampling is possible, the same (sample – resample) mass accounting framework can be utilized with potentially higher chances of representative sampling between time points. Incomplete dissolution of feedstock grains implies that some cations from feedstock remain within the solid phase of the postweathering soil-feedstock mixture. This does not constitute a methodological limitation: only cations that have been leached from the system are counted as weathered, consistent with quantifying realized CO_2_ removal.

Generally, EW deployments should assess *τ*_*j*_ values for all base cations to be used to estimate CDR. Because these will vary between base cations due to incongruent weathering and retention, setting the system of equations as an overconstrained system where a single τ_*j*_ value is optimized to fit observed trends for all base cations is not recommended. Some feedstocks may also contain mineral phases that are not expected to dissolve on time scales relevant for the EW deployment, which could be taken into account by modifying the composition of the hypothetical weathered feedstock residue endmember accordingly (see also section S1.1.3).

When only baseline and postweathering data are available (i.e., no postapplication sampling done), this endmember mixing approach is preferable to quantifying feedstock dissolution exclusively from the loss of cations compared to the initial soil-feedstock mix composition (eqs 1 and 2) because estimating this initial composition from postweathering measurements without knowing the exact mixing proportions (which may vary throughout a field) is nontrivial. Instead, the endmember mixing approach quantifies the dissolution fraction while also explicitly accounting for the enrichment of immobile elements due to feedstock loss from the system. Alternatively, sampling after feedstock addition (and again after weathering has occurred) can be used to resolve issues of mixing proportions. Comparing postweathering to postapplication data can often be favorable from a signal-to-noise perspective. Nevertheless, mobile element loss should still be calculated relative to a detrital element, even when not using the detrital element to calculate feedstock addition rates.^39,40,43,45^

#### 2.2.3 Analytical Solution for the Mass Transfer Coefficient τ_*j*_, Simplified Case

The endmember contributions reflect three unknowns. Hence, we set up two additional equations reflecting mass conservation of immobile elements as well as mobile base cations, respectively,

5 $${\left[j\right]}_{s}X_{s}\rho_{s}+{\left[j\right]}_{f}X_{f}\rho_{f}+{\left[j\right]}_{wf}X_{wf}\rho_{wf}={\left[j\right]}_{mix,t=n}\left(X_{s}\rho_{s}+X_{f}\rho_{f}+X_{wf}\rho_{wf}\right)$$

6 $${\left[i\right]}_{s}X_{s}\rho_{s}+{\left[i\right]}_{f}X_{f}\rho_{f}+{\left[i\right]}_{wf}X_{wf}\rho_{wf}={\left[i\right]}_{mix,t=n}\left(X_{s}\rho_{s}+X_{f}\rho_{f}+X_{wf}\rho_{wf}\right)$$

where ρ_*i*_ is the density of each respective endmember. Note that it is important to account for the impact of immobile element enrichment due to mass loss also for the composition of the hypothetical weathered feedstock endmember. These equations can be solved (see S1.1 for detailed derivation) to calculate the contribution of each endmember to the observed postweathering composition. We first develop a simplified case of this framework where we assume that the density and mobile element composition of the weathered feedstock endmember is equivalent to initial baseline soil composition

7 $${\left[j\right]}_{wf}={\left[j\right]}_{s}$$

and

8 $$\rho_{wf}=\rho_{s}$$

The immobile element composition of the weathered-feedstock endmember can be approximated by summing the immobile elements contained in a volume of feedstock equivalent to the sampled layer with those in an equivalent volume of background soil that replaces the fully weathered feedstock. The resulting composition thus represents a hypothetical endmember in which the entire feedstock volume of sample-layer depth has weathered completely, and its immobile elements have been retained within the soil that has replaced it within the reference volume. The sum of these immobile elements is then divided by the mass of the background soil sampled layer

9 [i]wf=ρsvsampledlayer[i]s+ρfvsampledlayer[i]fρsvsampledlayer=ρs[i]s+ρf[i]fρs=[i]s+ρfρs[i]f

where *v*_sampled layer_ corresponds to the sampled soil volume and *ρ*_*i*_ to the density of feedstock and soil.

In this simplified case, equations of endmember contributions and *τ*_*j*_ simplify to (see S1 for derivations)

10 $$X_{f}=\frac{\rho_{s}\Delta {\left[j\right]}_{s}}{\rho_{s}\Delta {\left[j\right]}_{s}-\rho_{f}\Delta {\left[j\right]}_{f}}$$

11 $$X_{wf}=\frac{\rho_{s}\left(\Delta {\left[j\right]}_{s}\Delta {\left[i\right]}_{f}-\Delta {\left[j\right]}_{f}\Delta {\left[i\right]}_{s}\right)}{{\left[i\right]}_{f}\left(\rho_{s}\Delta {\left[j\right]}_{s}-\rho_{f}\Delta {\left[j\right]}_{f}\right)}$$

12 $$X_{s}=1-X_{f}-X_{wf}$$

13 $$\tau_{j}=\frac{\Delta {\left[j\right]}_{s}\Delta {\left[i\right]}_{f}-\Delta {\left[j\right]}_{f}\Delta {\left[i\right]}_{s}}{\Delta {\left[j\right]}_{s}{\left[i\right]}_{f}+\Delta {\left[j\right]}_{s}\Delta {\left[i\right]}_{f}-\Delta {\left[j\right]}_{f}\Delta {\left[i\right]}_{s}}$$

where Δ­[*n*]_*x*_ is the difference between postweathering soil and initial endmember (*x* = s, f, wf) immobile or mobile element concentration (*n* = *i*, *j*), e.g.,

14 $$\Delta {\left[j\right]}_{s}={\left[j\right]}_{mix,t=n}-{\left[j\right]}_{s}$$

The simplified formulation of the SOMBA framework provides a pragmatic and robust means of estimating dissolution fractions when the composition of the weathered feedstock endmember cannot be independently constrained. This simplification not only reduces input requirements but also has the benefit that quantification of τ_*j*_ is invariant to soil and feedstock density—an important advantage given that these parameters are rarely well characterized in field-scale deployments. The resulting bias from this assumption is demonstrably small within realistic parameter ranges (section 3.3), making the simplified case both operationally efficient and scientifically defensible for most EW applications. For this reason, the further analysis developed in this study utilize the simplified framework, with the nonsimplified case laid out below primarily used to assess the bias in the simplified framework when the assumptions are not met.

#### 2.2.4 Analytical Solution of τ_*j*_, Nonsimplified Case

We furthermore derive the expression for *τ*_*j*_ without these simplifying assumptions (see S1.1.2 for full derivation and derivation of endmember contributions):

$$\tau_{j}=\frac{1}{1-\frac{\rho_{wf}}{\rho_{f}}\frac{\Delta {\left[i\right]}_{wf}-\frac{\Delta {\left[j\right]}_{wf}\Delta {\left[i\right]}_{s}}{\Delta {\left[j\right]}_{s}}}{\Delta {\left[i\right]}_{f}-\frac{\Delta {\left[j\right]}_{f}\Delta {\left[i\right]}_{s}}{\Delta {\left[j\right]}_{s}}}}$$ 15

Note that this final formulation of τ_*j*_ is invariant to soil density but still depends on the densities of the weathered and nonweathered feedstock endmembers. Full derivation for this equation as well as analytical solutions for the endmember contributions can be found in S1.

Importantly, if the composition of the weathered-feedstock endmember is adjusted to account for cation retention in topsoils, the equivalent amount of base cations must be deducted from the rock’s total CDR potential as defined by its initial composition. Because weathering is then quantified relative only to the fraction of cations that are truly mobile, any retained cations must be subtracted to avoid overestimating CDR (see also section 3.5)­

$$CDR_{pot,adjusted}=CDR_{pot}\left(1-f_{j,retained}\right)\approx CDR_{pot}\frac{{\left[j\right]}_{f}-{\left[j\right]}_{wf}}{{\left[j\right]}_{f}-{\left[j\right]}_{s}}$$ 16

where *f*_*j*__,retained_ is the fraction of cations retained upon weathering and CDR_pot_ the feedstock’s maximum CDR potential as estimated via the Steinour formulation from bulk composition.^58,59^ See section S1.1.3 for more details, as well as an approximation of the weathered feedstock composition in this case.

#### 2.2.5 Estimation of Initial Agronomic Parameters from Postweathering Samples

In addition to estimating feedstock dissolution, both the simplified and nonsimplified frameworks presented here can also be used to estimate the amount of initial feedstock as well as the preweathering feedstock-soil mix composition from the postweathering composition as well as baseline soil and feedstock data (for a detailed derivation see S1)­

17 $$a=\left(X_{f}+X_{wf}\right)v_{sampled\;layer}\rho_{f}$$

18 $${\left[j\right]}_{mix,t=0}=\frac{\rho_{s}X_{s}{\left[j\right]}_{s}+\rho_{f}\left(X_{f}+X_{wf}\right){\left[j\right]}_{f}}{\rho_{s}X_{s}+\rho_{f}\left(X_{f}+X_{wf}\right)}$$

19 $${\left[i\right]}_{mix,t=0}=\frac{\rho_{s}X_{s}{\left[i\right]}_{s}+\rho_{f}\left(X_{f}+X_{wf}\right){\left[i\right]}_{f}}{\rho_{s}X_{s}+\rho_{f}\left(X_{f}+X_{wf}\right)}$$

where *v*_sample layer_ is the volume of the sampled layer (per hectare if a is estimated per hectare).

#### 2.2.6 Implementation

Here, we supply Python code as well as an example use case. Generally, the relevant calculations are defined as functions in the Python file SOMBA.py, where the calculation of *τ*_*j*_ is defined in the functions SOMBA_tau and SOMBA_tau_simplified (for the nonsimplified and simplified frameworks). The code also contains additional functions to estimate preweathering and postweathering mix composition from deployment data (functions SOMBA_start, SOMBA_end and SOMBA_end_simplified; see supplement S1). In addition, SOMBA.py also contains the function SOMBA_tau_meta and SOMBA_tau_meta_simplified, which in addition to *τ*_*j*_ also return the individual endmember contributions as well as additional deployment parameters calculated from postweathering samples as defined below.

We provide four Python scripts; two (one each for simplified and nonsimplified case) that load input data and calculate the SOMBA parameters, and two that demonstrate the internal consistency of the framework presented here (see also supplement S1.5 and Figure S2). We also provide Excel templates that calculates the dissolution fractions for both the simplified and nonsimplified approaches. These templates may be used as a tool to analyze initial results, but ultimately thorough statistical investigation should always be based on statistical modeling.

### 2.3 Signal-to-Noise Analysis

Soils are heterogeneous both on small and large spatial scales,^60–67^ which may pose challenges for soil-based approaches to quantify rock powder dissolution in EW field trials.^36,54,56^ To assess the efficacy of the soil-based mass balance approach to quantify rock powder dissolution outlined here against the backdrop of soil heterogeneity, we conduct a signal-to-noise analysis grounded in soil and basalt data for EW field trials in US agricultural lands.

#### 2.3.1 Data Constraints

To use a representative basalt composition, we calculate the mean composition (in terms of base cations and Ti) of all basalts within the US that are contained in the GEOROC database.^68^ Soil element concentrations as well as representative soil heterogeneity on these parameters are based on two separate data sets. We use an existing data set of US soils^65^ to constrain the elemental composition of a large number of fields (only data classified as “Row Crops” and “Small Grains” as LandCover2 variable considered). Here, each sample is considered to represent the “true” composition of a field. The analysis uses Ca + Mg as *j* (basalt [*j*]_f_ = 3.11 mol kg^–1^) and Ti as *i* (basalt [*i*]_f_ = 0.206 mol kg^–1^). Because the SOMBA framework requires a clear difference in [*i*] and [*j*] between soils and rock powders,^36^ we only consider fields as suitable where both [*i*] and [*j*] are at least 5 times lower than US basalt (∼22%; *n* = 130; Figure S3). These data are used as “true” field compositions.

To constrain variance on field-level sample compositions resulting from spatial heterogeneity, we utilize a new data set of soil heterogeneity based on high-density spatial sampling (Table 1; Figure S4). This data set includes new ICP-MS soil composition measurements (residual phase after exchangeable cations were leached with 1 M ammonium acetate) from 5 field sites in the US with spatial sampling frequencies ranging from 0.6–19.8 samples ha^–1^ (7.1–39.6 pooled subsamples ha^–1^). For more information on sampling and analytical procedures, see supplement S2. We fit log-normal distributions to field data (using the Python scipy.stats module), and use fitted shape parameters representing the standard deviations (σ) of the underlying normal distribution to model in-field variance. The shape parameters corresponding to field data are shown in Figure S5, and uniform distributions between the range of observed shape parameters is used to generate synthetic σ values in Monte Carlo simulations.

**Table 1:** Information on the Field Sites Used to Constrain Spatial Heterogeneity in the Signal-to-Noise Analysis^a^

								**soil heterogeneity** (σ; log-normal)			
**site name**	**lat [deg]**	**lon [deg]**	**size [ha]**	**# samples**	**# pooled cores**	**sample density [ha^–1^]**	**core density [ha^–1^]**	**Ca**	**Mg**	**Na**	**Ti**
Site 1	45.3	–87.6	6.42	40	2	6.23	12.46	0.493	0.278	0.072	0.120
Site 2	42.3	–73.6	5.08	41	2	8.07	16.14	0.395	0.309	0.250	0.288
Site 3	31.3	–84.4	2.02	40	2	19.80	39.60	0.582	0.218	0.630	0.264
Site 4	35.8	–78.2	42.44	25	12	0.59	7.07	0.519	0.523	0.510	0.154
Site 5	35.8	–78.2	26.85	38	12	1.42	16.98	0.355	0.687	0.391	0.177

a The number of pooled cores corresponds to the number of subsample cores that were combined for each measured sample. Soil heterogeneity refers to the σ of log-normal fits to soil concentration distributions normalized to the field mean such that the resulting distribution has a mean of 1 (Figure S4). Site names are anonymized, and location data are rounded to one decimal degree to protect farmer privacy.

#### 2.3.2 Statistical Modeling

The signal-to-noise analysis developed here predicts the efficacy of detecting feedstock dissolution based on hypothetical application amounts and dissolution fractions (*τ*_*i*_) and a paired sampling approach in a series of Monte Carlo simulations based on the following logic. For each modeled τ_*j*_ value, application amount, and sampling frequency (1–20 samples ha^–1^), we do the following:

1. Generate the number of samples to be simulated for each field from the product of sampling frequency and a simulated field size, ranging from 10 to 100 ha (uniform distribution). Within the US, most farms are smaller than 72 ha, but most farmland is in farms that are larger than 2000 ha,^69,70^ such that the values generated here represent a conservative choice.
2. Generate a set of baseline soil samples for each field based on log-normal distributions where the variance is constrained from fits to empirical data (Figures S4 and S5), and the generated log-normal sample distributions scaled to ensure the expected population mean is the same as the “true” field mean (see also supplement S2.3).
3. Calculate the “true” postweathering composition for each baseline sample, based on deployment parameters (using functions SOMBA_start and SOMBA_end_simplified)—reflecting a paired sampling approach.
4. Generate variance around the “true” postweathering compositions as in (1), assuming both (i) an additional variance term equal to the baseline variance (σ, representing a conservative scenario with 100% added variance) and (ii) a reduced additional variance of 50% σ, reflecting the potential improvement achievable through spatially paired sampling. While this reduction is somewhat arbitrary, the variance of paired postweathering samples compared to baseline samples can be empirically evaluated in future deployments once pre- and postapplication data sets of sufficient density become available. Both scenarios add additional variance compared to baseline samples.
5. Randomly generate uncertainty on feedstock composition from 5–10% (uniform distribution, i.e., σ values of 0.05 to 0.1 for generated log-normal distributions). To reflect increasing thoroughness of the sampling approach, as soil sampling frequency increases from 1 to 20 samples ha^–1^ we also increase the number of total samples that the composition of the feedstock endmember is calculated from (from 1 to 20 samples).
6. Calculate the average baseline, postweathering soil-feedstock mix, and feedstock composition based on the generated samples, each called one “realization”.
7. For each realization, calculate the dissolution fraction (based on SOMBA_tau_simplified) and calculate the absolute difference compared to the true dissolution fraction (which is assumed *a priori*).
8. Repeat this procedure one hundred times (100 realizations) and calculate the average error on *τ*_*j*_ over all fields and realizations. This average error represents the expected error on *τ*_*j*_ if applying this framework based on data-constrained soil heterogeneity and representative US soil composition.

## 3 Results and Discussion

### 3.1 Calculated Dissolution Fractions outside of the Mixing Triangle

The framework introduced here should only be applied when the postweathering composition of the feedstock-soil mixture falls within mass balance constraints—the mixing triangle defined by the soil, feedstock, and hypothetical weathered feedstock residue endmembers (Figure 1d). For this to be the case, the application amount, dissolution fraction, and the difference in soil and feedstock immobile element as well as base cation content need to be sufficiently large^36,56^ such that weathering of rock powder results in a statistically significant signal. If a significant portion of the samples in the sample-resampling approach fall outside of the mass balance constraints, it is most likely a sign that the sampling strategy was not optimized for capturing the underlying spatial variation in soil chemistry and/or that soil and feedstock compositions were too similar.^36^ Given that soil sampling methods have been discussed in detail in numerous places^30,56,71^ we will not address them here.

For the framework developed here to produce compositions outside of the endmember mixing triangle, at least one of the endmember contributions to the postweathering sample would need to be negative. Because *τ*_*j*_ is computed as the contribution of the weathered feedstock residue endmember relative to the sum of the same endmember and the residual feedstock endmember contributions (eq 4), if either of these contributions is negative the denominator of this fraction can approach 0, which causes instability outside of the mixing triangle. This is demonstrated in Figure 2, where *τ*_*j*_ is shown as a function of [*i*] and [*j*] for two hypothetical soil and feedstock compositions. As evident from Figure 2, outside of the mixing triangle *τ*_*j*_ tends to increase to unrealistically large absolute values. To the left of the soil endmember (indicating a hypothetical negative amount of feedstock), reasonable but unphysical *τ*_*j*_ can be achieved as a result of noise. These observations suggest: (1) when applied to field settings, this framework requires thorough statistical investigation to ensure that the postweathering composition is significantly different to pure soil and preweathering soil-feedstock mixtures. This requires, for example, Monte Carlo-type statistical approaches in which the uncertainty introduced by all parameters (including potential corrections for control site trends to baseline data) is fully propagate d into final estimates;^54^ (2) although individual samples may fall outside of the mixing triangle as a result of soil heterogeneity even if there is a robust signal overall, because mixing compositions outside the mixing plane are unstable, *τ*_*j*_ should always be computed based on sample population averages rather than from the average of *τ*_*j*_ calculated for individual samples.

**Figure 2:**
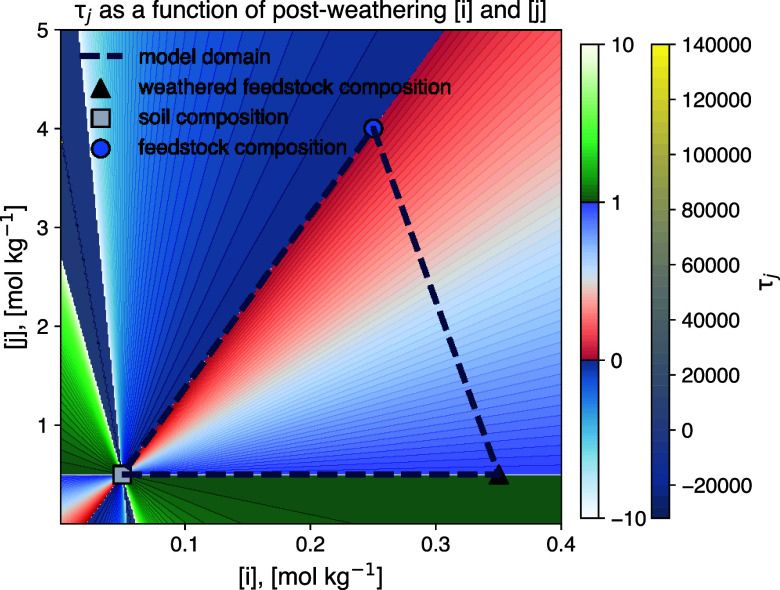
Quantified feedstock dissolution fractions (τ_*j*_) for a hypothetical soil and rock powder for a range of immobile element (*i*) and base cation (*j*) concentrations. The framework developed here should only be applied within the mixing triangle set out by baseline soil, rock powder, and the hypothetical weathered feedstock residue endmember. Outside of this domain, the results of the framework are unstable, and absolute values can approach infinity because negative contributions of endmembers can cause the dominator of eq 4 to approach 0. Generally, the framework developed here should only be applied when postweathering soil-feedstock mix composition robustly falls within the mixing triangle.

### 3.2 Non-Self-Averaging Behavior

The framework presented here is non-self-averaging; i.e., it does not average linearly across samples. This means that calculating *τ*_*j*_ for each individual sample and then taking the average does not give the same result as calculating *τ*_*j*_ based on the sample population average *i* and *j* concentrations. This phenomenon is particularly acute when some samples fall outside of the mixing triangle.

We demonstrate this behavior with a simple simulation (Figure S14 and Table 2). We calculate the true pre- and postweathering soil-feedstock mixture compositions for two hypothetical deployments (250 t ha^–1^, *τ*_*j*_ of 0.5 and 50 t ha^–1^, *τ*_*j*_ of 0.25) and endmember compositions (using the Python functions SOMBA_start and SOMBA_end_simplified). For the calculated postweathering composition, we simulate a set of samples based on assumed soil heterogeneities (here implemented as normal distributions with relative standard deviations of 25% and 10%, respectively) such that these two sets correspond to exemplary low- and high-resolvability deployments. For the first deployment many samples fall outside of the mixing triangle (Figure S14c). For both populations, *τ*_*j*_ as calculated from the average of each individual sample *τ*_*j*_ is not the same *τ*_*j*_ calculated from the population mean *i* and *j* concentrations (Table 2), with an extreme difference for the first low-resolvability scenario. This is important to consider in statistical modeling of postdeployment data, where Monte Carlo approaches (incl. bootstrapping) should always first calculate population means based on sample chemical compositions before calculating *τ*_*j*_ for a specific average model composition, rather than statistically resampling from distributions of sample *τ*_*j*_.

**Table 2:** Realized Sample Compositions and Their Calculated τ_*j*_ as well as Population Average Sample Composition and Its τ_*j*_ for Two Hypothetical EW Deployments (50 t ha^–1^, τ_*j*_ = 0.25, SD of Randomly Generated Soil Compositions = 25% as well as 250 t ha^–1^, τ_*j*_ = 0.5, 1SD = 10%)

**example deployment 1,** **50 t ha** ^**–1**^ **, τ**_***j***_ **= 0.25, SD = 0.25**			**example deployment 2,** **250 t ha** ^**–1**^ **, τ**_***j***_ **= 0.5, SD = 0.1**		
***i*** **[mol kg^–1^]**	***j*** **[mol kg^–1^]**	**τ**_***j***_	***i*** **[mol kg^–1^]**	***j*** **[mol kg^–1^]**	**τ**_***j***_
I. Random Samples (Postdeployment Composition)					
0.033	0.569	1.300	0.089	0.707	0.652
0.054	0.777	–1.525	0.082	0.808	0.400
0.053	0.389	8.379	0.093	0.698	0.694
0.076	0.522	0.940	0.085	0.764	0.515
0.078	0.365	1.374	0.096	0.924	0.413
0.035	0.723	2.392	0.096	0.893	0.452
0.061	0.564	0.631	0.072	0.846	0.078
0.051	0.455	6.083	0.079	0.880	0.217
0.063	0.641	0.313	0.096	0.751	0.635
0.066	0.623	0.506	0.093	0.817	0.519
random sample average τ_*j*_		2.039			0.457
II. Sample Average [*i*], [*j*], and Related τ_*j*_					
0.057	0.563	0.433	0.088	0.809	0.481
III. Calculated True Composition					
0.057	0.591	0.25	0.089	0.802	0.5

### 3.3 Sensitivity Analyses

To evaluate the robustness of the SOMBA framework under relevant field conditions, we conducted a suite of sensitivity analyses targeting the impact of immobile element enrichment as well as key input uncertainties and structural assumptions. Detailed procedures and extended results are provided in supplement S3.

One of the major advances of the framework presented here is the handling of immobile element enrichment in soils due to rock powder mass and volume loss. Accounting for this effect is clearly important: at low feedstock-to-soil [*i*] concentration ratios (*r*_*i*_) and *τ*_*j*_ values, weathering fractions can be overestimated by more than 50% otherwise (Figure 3a). While the absolute enrichment of immobile elements (Δ­[*i*]) scales with application amount (Figure S6a–c), its effect on the recovered dissolution fraction (τ_*j*_) is invariant to application rate (Figure S6d–i), because both the mobile and immobile inventories scale proportionally with added feedstock mass. At more expected weathering rates, neglecting this enrichment can bias recovered τ_*j*_ values by up to 10–20%. Therefore, this process be taken into account—even when Δ­[*i*] itself is too small to be directly resolved from background variability.

**Figure 3:**
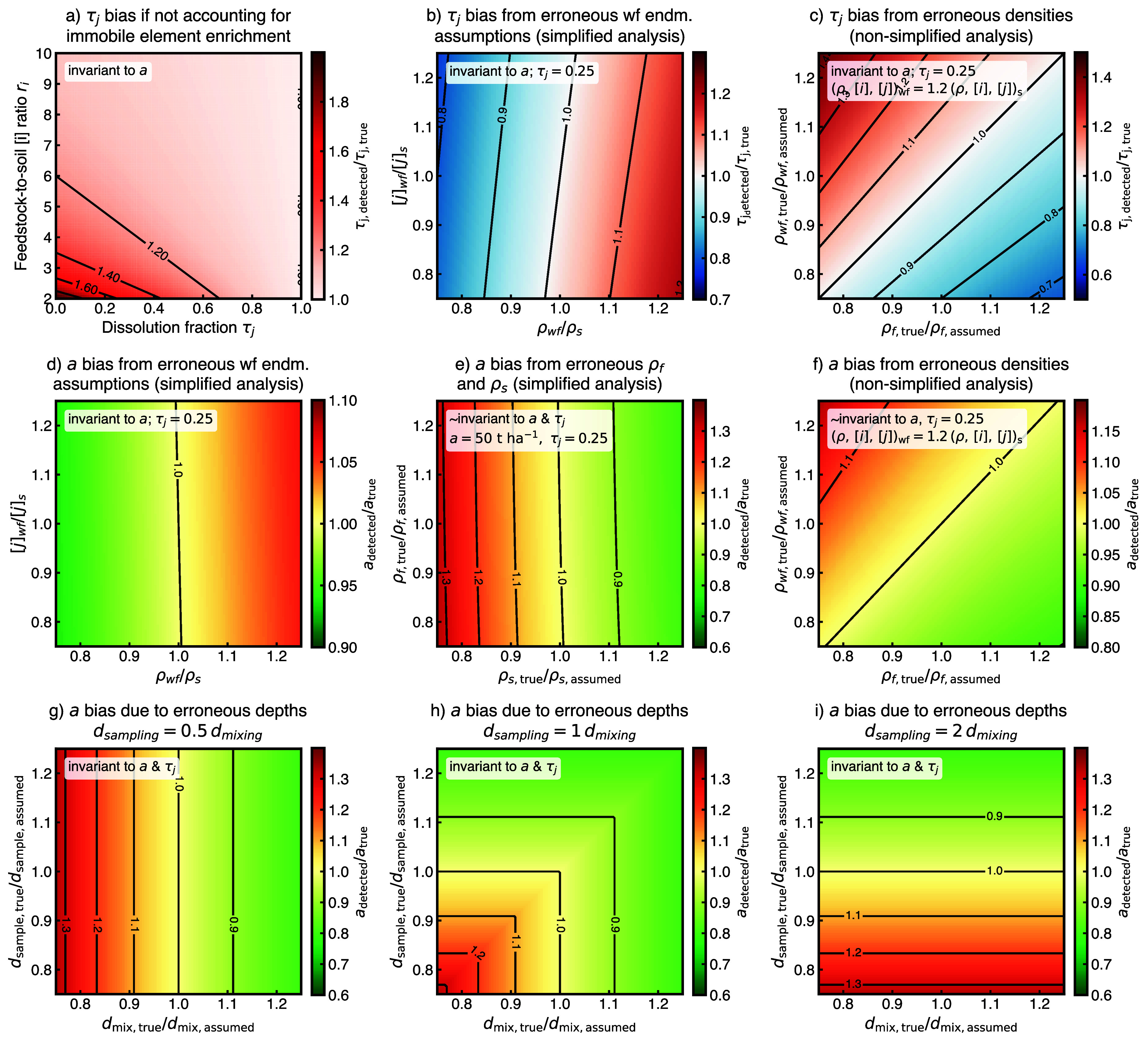
Sensitivity of the SOMBA framework to immobile element enrichment and input parameter uncertainties. Panel a illustrates how immobile element enrichment caused by feedstock mass loss biases estimated τ_*j*_ values when uncorrected, leading to systematic overestimation at low compositional contrasts between soil and rock powder (*r*_*i*_) and low dissolution fractions (τ_*j*_). Panels b and c show the resulting bias on τ_*j*_ when (b) the simplified SOMBA assumptions are violated—i.e., when the weathered feedstock density and mobile element concentrations deviate from those of the baseline soil—and (c) when feedstock and weathered-feedstock densities (ρf, ρwf) are mischaracterized in the nonsimplified framework. Panels d–f depict biases in the estimated rock powder application amounts. In the simplified case, violations of the underlying assumptions (d) or errors in soil and feedstock densities (ρs, ρf; e) can lead to underestimation of application amounts, with underestimating ρ_s_ producing the strongest bias (up to ∼30%). This effect does not exist for τ_*j*_ as soil and feedstock densities cancel out (see eq 13). In the nonsimplified framework (f), mis-specification of ρ_wf_ and ρ_f_ introduces smaller, systematic biases that increase with τ*_j_* (Figure S11). Panels g–i show the influence of mischaracterized sampling and mixing depths (*d*_sampling_, *d*_mixing_) on recovered rock application amounts for three scenarios: *d*_mixing_ = 0.5*d*_sampling_ (g), *d*_mixing_ = *d*_sampling_ (h), and *d*_mixing_ = 2*d*_sampling_ (i). The scenario where sampling and mixing depths are equivalent is the most robust to overestimation, whereas mismatched depths can cause apparent application rates to deviate by ∼30%. Notably, these depth inaccuracies do not affect estimated τ_*j*_ values. Detailed sensitivity procedures and additional simulations are provided in Supplementary Section S3.1 and Figures S6–S11.

In theory, one could use an immobile element that is strongly depleted in feedstock relative to soil to calculate feedstock addition from the depletion of *i* in the mixed sample. However, the utility of this approach is limited as the vector caused by the enrichment of immobile elements through feedstock mass loss will align with the mixing line, making it difficult to discern significant trends (Figure S13). Hence, the framework discussed here should only be applied when immobile element concentrations in feedstock are greater than those present in background soil. In contrast, calculated *τ*_*j*_ may be erroneously small if “immobile” elements used as a proxy for feedstock addition are in fact mobilized, as is observed for example for Ti and Zr in some extremely weathered and cation depleted soils.^72–74^ This phenomenon would cause underestimation of the amount of initially added feedstock when immobile elements are used as a proxy for feedstock addition, resulting in estimates of base cation loss and dissolution fractions that would be biased low. While this is of less concern than potentially overestimating weathering for the purpose of verifying CDR credits, practitioners should validate the immobility of their chosen proxy elements—preferably by comparing treated and control plots—particularly in acidic or highly weathered soils. Demonstrating immobility is therefore in the interest of deployers to avoid under-crediting and critical for ensuring methodological credibility.

We additionally assess the sensitivity of both the simplified and nonsimplified frameworks to uncertainties in the primary input parameters and in the underlying assumptions used to calculate *τ*_*j*_ and *a*. In the simplified case, the estimation of *τ*_*j*_ within SOMBA is independent of soil and feedstock density, and in the nonsimplified case, independent of soil density alone (see sections 2.2 and S1). Quantification of *τ*_*j*_ is likewise unaffected by inaccuracies in sampling or mixing depth characterization, as the framework relies on relative changes in mobile and immobile element concentrations and is invariant to such concentrating or diluting processes (including erosion). As a result, the framework remains relatively robust for quantifying initial rock-powder weathering. However, τ_*j*_ becomes biased when the assumptions of the simplified formulation are violated, yet the simplified framework is still applied (Figure 3b; Figure S7). In the nonsimplified framework, mis-estimation of feedstock and weathered-feedstock endmember densities similarly biases *τ*_*j*_ (Figure 3c; Figure S10). Across realistic parameter ranges, both sources of error can introduce systematic over- or underestimation of *τ*_*j*_ of up to approximately 25%.

Quantification of the initially added rock powder is more sensitive to errors in input parameters, particularly endmember densities and the characterization of mixing and sampling depths. While violations of the simplified framework’s assumptions have only a marginal influence on the recovered application amounts (Figure 3d; dependence on *τ*_*j*_ shown in Figure S8), underestimating soil density (*ρ*_s,true_ > *ρ*_s,assumed_) can lead to derived rock application rates that are approximately 30% too low (Figure 3e; Figure S9). In the nonsimplified framework, inaccurate specification of feedstock or weathered-feedstock densities introduces smaller but systematic biases that increase with *τ*_*j*_ (Figure 3f; Figure S11). We further evaluate the effects of mischaracterizing sampling and mixing depths on estimated rock powder application amounts under three scenarios in which the sampling depth is half, equal to, or twice the true mixing depth (Figure 3g–i). For the first case, application amounts can be substantially (∼30%) overestimated when the actual mixing depth is less than the targeted value, whereas for the latter case the same is true when sampling depth is too low compared to the targeted value. When the targeted sampling and mixing depths are equivalent, both conditions must be simultaneously met to yield overestimation (both sampling and mixing depth less than target value). We therefore recommend that sampling depth be matched as closely as possible to the true mixing depth to avoid overestimation.

While these factors can influence the quantification of applied rock powder, we emphasize that the presented framework remains relatively robust within the tested parameter space and, to our knowledge, represents an implementable approach capable of estimating rock application rates from postweathering samples. This can provide essential information for verification purposes for deployments in carbon crediting programs. Application amounts could also be constrained independently at the field scale when acquisition and spreading records are available and well-documented. The calculation of τ_*j*_ is robust within the examined parameter ranges for the simplified framework, with the effects of potential errors in elemental concentration measurements addressed in the following section on signal-to-noise analysis (section 3.4). Nonetheless, the sensitivity of results to parameters such as the densities of weathered rock powder and the accuracy of sampling or mixing depth characterization highlights the need for targeted laboratory studies (e.g., refining the composition of the weathered-feedstock endmember) and for technological innovation in sampling equipment.

### 3.4 Signal-to-Noise Analysis

The framework presented here can only yield accurate estimates of rock powder dissolution in soils when weathering signals can be picked out against background soil heterogeneity.^36,53,54,56^ Here, we assess signal-to-noise in the updated framework by estimating the average error on detected dissolution fractions practitioners would observe based on specific deployment choices and spatial sampling frequencies. This analysis is based on a novel in-field data set of high spatial density (0.6–19.2 ha^–1^; Table 1), which are used to simulate in-field heterogeneity.

The average error on detected dissolution fractions decreases with increasing sampling frequency, application amounts, and dissolution fractions (Figure 4), which is consistent with previous investigations.^36,56^ Based on conservative estimates of the ability of paired sampling to limit postweathering variance (σ scenario; Figure 4a,b); the signal-to-noise analysis suggests that when cumulative application amounts exceed 100 t ha^–1^, expected errors are on average <15% when sampling frequencies exceed 10 samples ha^–1^. At higher dissolution fractions (*τ*_*j*_ = 0.5), average errors below 10% are possible with high sample density. However, meeting these conditions is not possible in many deployments, suggesting that this simplistic sampling strategy generally not advisible for short-term monitoring of individual fields. If a paired-sampling approach is implemented effectively the average error decreases significantly (σ/2 scenario; Figure 4c,d). The implementation of postweathering variance (100% and 50% additional σ relative to baseline) is necessarily simplified, with both modeled cases imposing significant additional variance. Pooling sampling on geolocated samples with a postapplication baseline will also minimize this variance. However, in practice, the relative variances of baseline and postweathering samples can be empirically constrained from field-deployment data. Monte Carlo simulations should therefore be tailored to site-specific conditions to quantify uncertainty in detected dissolution fractions and assess the applicability of the SOMBA framework under real-world variance structures.

**Figure 4:**
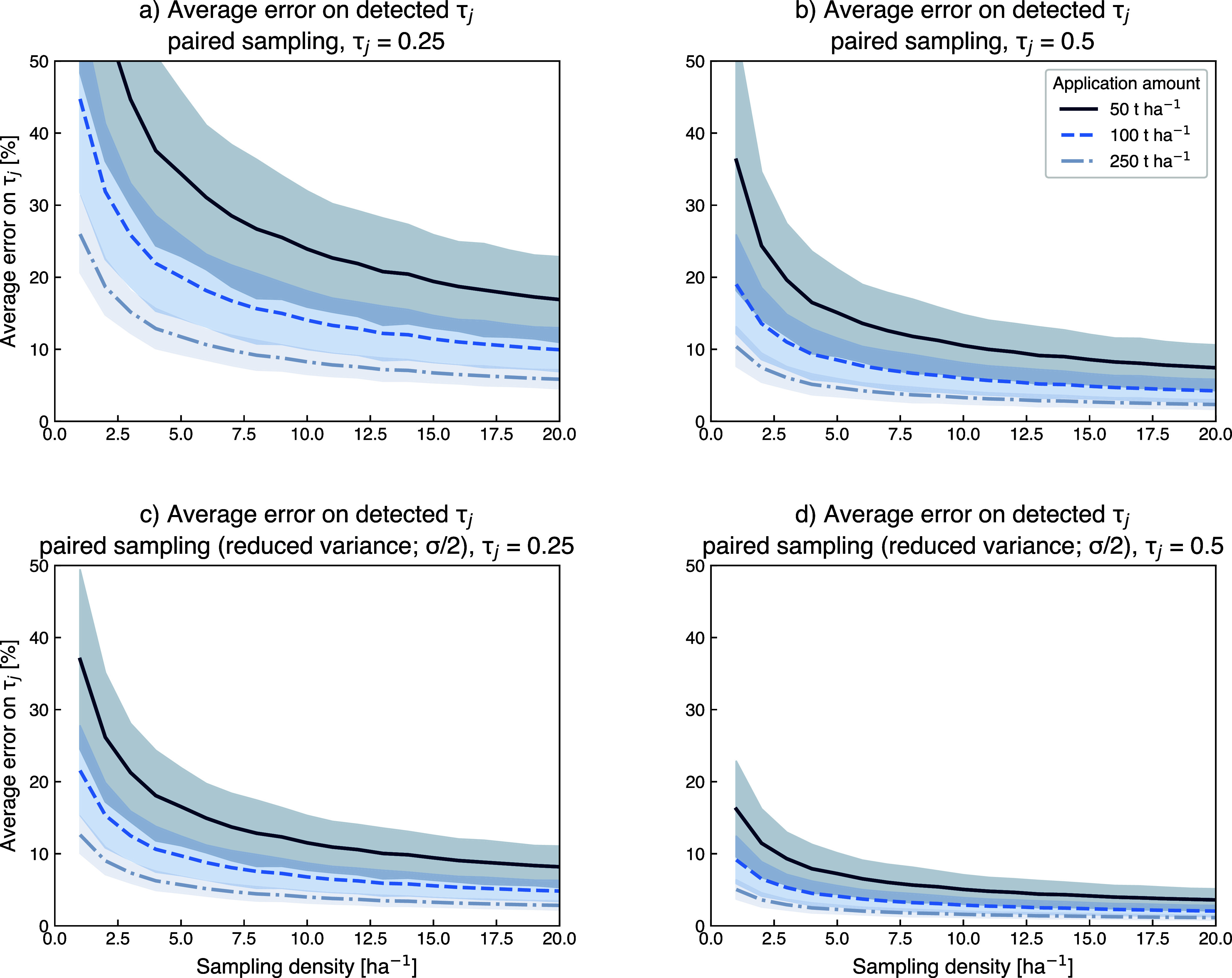
Average errors on detected dissolution fractions for two simulated mass transfer coefficients (τ_*j*_ = 0.25 in a and c, τ_*j*_ = 0.5 in b and d). The top row shows simulations where the variance imposed onto paired samples is equivalent to the variance of initial baseline samples. Because is likely an overestimate for accurate sample and resample strategies, the lower row shows the same simulations based on reduced variance for resampled sample composition (σ/2). The simulations are based on compositions of US soil^65^ and basalt^68^ considering soils with base cation and Ti concentrations at least 5 times lower than basalt. The simulated in-field soil heterogeneity is based on the novel field trial data set presented in Table 1.

In the context of sampling densities, it should be noted that if the spatial distance between subsample cores used to pool samples is larger than the spatial wavelength of soil heterogeneity, required high sample frequencies can also partially be achieved by pooling samples, as is commonly practiced in most agronomic soil sampling protocols.^75^ Additionally, there are well established methods for characterizing contaminants in soils and other particulate media.^76–81^ A key aspect of these methodologies, such as Incremental Sampling Methods (ISMs), is that they acknowledge a-priori that soil components are distributed unevenly at the scales of interest relevant to signal detection. When implemented properly, incremental sample pooling and averaging strategies result in highly representative soil data at the field scale,^76–81^ with high numbers of pooled subsamples of sufficient individual mass having a higher utility compared to more measured samples reflecting less pooled cores, not least because the former tend to be normally distributed. We assert that if the mean and variance of a field or fields can be well established, averaged mixing models such as SOMBA can be utilized with confidence commensurate to the established population statistics.

This analysis is based on a subset of samples for which SOMBA are suitable for MRV, here operationally defined as soil [*i*] and [*j*] being at least 5 times below US-average basalt composition.^68^ Approximately ∼22% of all agricultural soils contained in the used geochemical soil database^65^ fulfill this condition. This fraction strongly increases when all soils with concentrations at least 2 times lower are considered (*n* = 594 out of 614 samples; ∼95%), primarily due to more samples fulfilling the Ti cutoff (Figure S15). When the same signal-to-noise analysis is applied to this larger set of fields the average error on detected mass transfer coefficients is larger (Figure S16). This is expected based on lower soil-feedstock compositional differences.^36^ Note also that the modeled deployment size (10–100 ha) has a direct impact on the signal-to-noise analysis as the number of generated samples is the product between field/deployment size and sampling frequency. While SOMBA is therefore not universally applicable, it remains a powerful tool for the subset of croplands where sufficient compositional contrast exists, and its utility can be further expanded through aggregated or regional monitoring approaches.^57^

Regardless of the potential to optimize signal detection at the field scale, quantify dissolution fractions at the aggregate level over multiple deployments rather than for individual field sites, as has for example been demonstrated for the quantification of changes in soil organic carbon stocks, is an obvious and essential way to increase both accuracy and precision of this approach.^20,57,82^ The potential for variability in geochemical signatures at the field scale also makes it critical that fields in carbon removal projects not be filtered based on observed geochemical signatures—specifically, absence of signals for weathering should not be used as grounds for filtering field data.

Our signal-to-noise analysis also demonstrates that SOMBA can produce robust estimates of rock powder dissolution under specific conditions. Nevertheless, resolvability also depends on the decisions made in practice with respect to acceptable uncertainty. While achieving an error of less than, e.g., 10% for 90% of the realizations^56^ is challenging in most settings unless application rates are high, larger uncertainties can still be acceptable in the context of crediting CDR if crediting is done at lower bounds of uncertainty.^30^ In addition, it is important to keep in mind that this approach requires an adequate difference in soil and feedstock composition. A signal-to-noise analysis for a given feedstock should hence only be based on the subset of soils that are potential targets for robust signals for this MRV approach, rather than through approaches that group all signals together regardless of soil suitability. Furthermore, it is important to note that what is relevant for this framework is the total application *amount*, not the annual *rate*. Hence, settings for which feedstock dissolution may not be resolvable initially can become resolvable over time through the gradual increase of cumulative application amounts as well as increases in the dissolution fraction over time.

### 3.5 Additional Assumptions and Limitations

One key assumption that is made in the signal-to-noise analysis is that baseline soil [*i*] and [*j*] does not change with time, and that therefore as long as sampling and spatial heterogeneity is correctly accounted for, a change in [*i*] and [*j*] can be solely attributed to feedstock addition and dissolution. This assumption may not always hold in cases where weathering of a labile constituent of the soil, aeolian deposition, or other process might unexpectedly result in loss or gain of elements in the soil. Changes through time in soil [*i*] and [*j*] in controls that cannot be explained by sampling practice and spatial heterogeneity should be factored into estimates for feedstock weathering generated using SOMBA; and results treated with caution where a mechanistic understanding of the elemental concentration change of the system cannot be found.^29^

We have not included baseline trend corrections in the signal-to-noise analysis presented here due to a lack of data on covariance for temporal trends in adjacent fields. Any simulation would hence depend more on our assumptions than realistic processes. As has been demonstrated, e.g., for soil organic carbon monitoring,^82^ including such a correction would increase average detection errors but not systematically change trends relating to different application amounts and sampling protocols. The absence of such data for EW currently represents a limitation of this study and may constitute an additional source of uncertainty that future work should explicitly address. Importantly, we suggest that for the purpose of crediting, cation losses from control sites should be deducted from treatment site EW signals, but that control site gains in base cations must not be used to increase weathering signals from treatment sites unless the cation gain in control site composition can be explained by known manipulations that have also occurred on treatment sites (e.g., manure input, etc.).

The analysis presented here assumes that mobile base cations are transported out of the sampled layer following feedstock weathering, although in reality some may be retained, particularly on the time scales relevant to EW. Both the simplified and nonsimplified SOMBA frameworks account for this behavior in different but physically consistent ways (see section S1.1.3 and Figure S1 for a detailed discussion). In the simplified case, cation retention via secondary phases formation or presence of unreactive feedstock components means that postweathering compositions never reach the base of the mixing triangle and τ_*j*_ does not reach 100%, yielding accurate weathering estimates relative to the cations actually released rather than implying removal where none occurred. In the nonsimplified framework, retention is incorporated through the adjusted composition of the weathered-feedstock endmember, allowing τ_*j*_ to physically approach 100%. Importantly, when estimating CDR from *τ*_*j*_, the corresponding CDR potential of the feedstock must then be reduced by the fraction of cations retained to prevent overestimation of CDR. Thus, this becomes a question of accounting—either one corrects for this retention and defines 100% weathering relative to the adjusted weathered feedstock composition (nonsimplified case), or one keeps the original feedstock basis and accepts that weathering fractions will remain below 100% (simplified case). In both cases, the SOMBA framework yields conservative, physically consistent estimates of realized weathering.

## 4 Implications

We have presented an updated framework for using SOMBA to quantify rock powder dissolution in EW field settings and provide Python code for implementing this framework. The updated framework explicitly accounts for the enrichment of immobile elements in topsoils due to feedstock mass loss.^39,45^ Failing to account for these processes can cause detected dissolution fractions to be overestimated. A comprehensive sensitivity analysis further shows that for the quantification of τ_*j*_, many density terms cancel analytically, making the framework inherently robust to uncertainties in bulk density and mixing depth. Where such parameters do not cancel—particularly for estimates of applied rock mass—resulting biases remaining small across realistic parameter ranges.

We suggest that the framework presented here should only be used when postweathering sample compositions fall robustly within the mass balance constraints defined by the endmember mixing approach. Solutions can be unstable outside of this parameter space, which may yield dissolution fractions that are unphysically high or low and should not be used to estimate CDR. The code presented here generates sensible dissolution fractions when the postweathering composition falls within the mass balance dictated mixing triangle. However, this does not necessarily mean that this signal can be resolved statistically. It is the responsibility of practitioners to thoroughly investigate the statistical significance of changes in soil compositions and deduced rock dissolution parameters, for example through stochastic simulations that propagate uncertainties pertaining to all relevant parameters^54^ including resulting from trends in control sites and through downsampling statistical tests. Sampling protocols should generally be defined *a priori*, and be informed by desired sampling power.^83^ Lastly, we suggest that the framework should be applied to calculate dissolution fractions based on the sample population mean, rather than for each individual sample. This consideration will change the statistical modeling of weathering dynamics, e.g., via Monte Carlo simulations. Future studies should include incremental sampling strategies at predefined field and subfield scales. In effect, averaging strategies should be fit-for-purpose and built in concert with the soil sampling procedures that researchers or EW suppliers design. We would also like to stress that alternative parametrizations of SOMBA are feasible and may be advantageous for certain settings.

Our signal-to-noise analysis suggests that field-level quantification of rock powder dissolution based on SOMBA is possible when application amounts, dissolution fractions, soil-feedstock compositional differences, and sampling frequencies are sufficient. Our analysis suggests that SOMBA can be a useful tool in tracking weathering rates, but it must be acknowledged that this approach will not work in all settings and will typically require higher sampling densities than those currently being implemented in academic and commercial deployments. Signal emergence can furthermore be optimized using tailored sampling strategies^56^ as well as feedstock-soil-matching.^36^ Given the potential for signal-to-noise issues to be present at the field scale, carbon accounting will be more robust when aggregating at a regional scale.^20,57,82^ The potential for variability in geochemical signatures at the field scale also indicates that it is critical that fields in carbon removal project not be filtered based on observed geochemical signatures (e.g., absence of signals for weathering).

## Supplementary Material



## Data Availability

SOMBA Python code and Excel templates for calculating dissolution fractions and deployment parameters are available at 10.5281/zenodo.15696933.

## Notes

Additional derivations of the soil mass balance framework (S1); soil sampling and analytical procedures (S2.1); field soil composition data sets and heterogeneity characterization (S2.2); sensitivity analyses of framework assumptions; Monte Carlo signal-to-noise methodology (S3); and supplementary figures of deployment parameter effects (S4) (PDF)
