# Supplementary material for: An Updated Framework and Signal-to-Noise Analysis of Soil Mass Balance Approaches for Quantifying Enhanced Weathering on Managed Lands
Source: Environ Sci Technol. 2025 Nov 20;59(49):26440–53. doi: 10.1021/acs.est.5c08303 (PMC12713775; doi:10.1021/acs.est.5c08303)
Supplement: Supplementary file 1 [file es5c08303_si_001.pdf]

Supplementary information to

An updated framework and signal-to-noise analysis of soil mass balance approaches for quantifying enhanced weathering on managed lands

Tim Jesper Suhrhoff[1,2 *], Tom Reershemius[3, 2], Jacob S. Jordan[4], Shihan Li[5], Shuang Zhang[5], Ella Milliken[2], Boriana Kalderon-Asael[2], Yael Ebert[2], Rufaro Nyateka[2], Jake T. Thompson[2], Christopher T. Reinhard[6], Noah J. Planavsky[2,1]

[1] Yale Center for Natural Carbon Capture, Yale University, New Haven, Connecticut 06511, USA

[2] Department of Earth and Planetary Sciences, Yale University, New Haven, Connecticut 06511, USA

[3] School of Natural and Environmental Sciences, Newcastle University, Newcastle upon Tyne, England NE1 7RU, United Kingdom

[4] Mati Carbon, Houston, Texas 77019, USA

[5] Department of Oceanography, Texas A&M University, College Station, Texas 77843, USA

[6] School of Earth & Atmospheric Sciences, Georgia Institute of Technology, Atlanta, Georgia 30332, USA

* corresponding author: timjesper.suhrhoff@yale.edu

Python code and Excel templates for the soil mass balance framework can be found here:

Content of this file:

S1: Additional derivations of the soil mass balance framework (incl. Figure S1 & Figure S2)

S2: Soil data (incl. Figure S3 to Figure S5)

S3: Impact of feedstock mass loss on base cation and immobile element concentrations (incl. Figure S6 & Figure S13)

S4: Impact of soil composition on signal-to-noise analysis (incl. Figure S14 & Figure S16)

S1 Additional derivations of the soil mass balance framework

This supplement contains all derivations relating to section 2.2 of the manuscript. In addition to quantifying rock powder dissolution and deployment parameters based on post-weathering soil sample composition (see section 2.2 of the main text), we also present a framework to calculate expected pre- and post-weathering compositions for soil-feedstock mixtures. The associated Python functions are included in the Python file SOMBA.py and were used in the analyses presented here. The code can be assessed here:
https://doi.org/10.5281/zenodo.15696933

S1.1 Derivation of soil mass balance framework

We continue from section 2.2 after the introduction of the hypothetical weathered feedstock endmember. The composition of this hypothetical endmember is defined to be the composition that a layer of soil would have after a layer of pure feedstock (corresponding to the soil sampling depth, d_{sample}) has dissolved.

Since cations are assumed to be lost from the system, it has the same cation content as an equivalent layer of pure soil, i.e.:

$$[j]_{wf} = [j]_s \qquad \text{S1}$$

where the subscripts *wf* and *s* correspond to weathered feedstock residue and baseline soil (and *f* denotes pure feedstock in the following equations). The immobile element concentration of this endmember is given by summing the amount of immobile elements in the fully weathered feedstock as well as the soil that has replaced the feedstock in the reference volume, and dividing by the system mass after weathering (assumed to be the same as background soil mass/density for the equivalent soil volume):

$$[i]_{wf} = \frac{\rho_s v_{sampled\ layer}\,[i]_s + \rho_f v_{sampled\ layer}[i]_f}{\rho_s v_{sampled\ layer}} = \frac{\rho_s[i]_s + \rho_f[i]_f}{\rho_s} = [i]_s + {}^{\rho_f}/_{\rho_s}\,[i]_f \qquad \text{S2}$$

where $v_{sampled\ layer}$ corresponds to the sampled soil volume and ρ to the density of feedstock and soil. We make the assumption that post-weathering density is equivalent to background soil density (see also eq. S6b) within the frame of reference assuming no change in porosity, though we note that this ignores weathering congruency and compositional difference between the soil parent material and the rock feedstock applied.

Assuming mass and volume conservation, this endmember mixing approach can be described by a system of equations such that each endmember contributes a volume proportion (*X*) to the observed post-weathering composition, which together sum to unity:

$$X_s + X_f + X_{wf} = 1 \quad \text{S3}$$

Because in practical field sampling based on constant soil sampling depths, a system of constant *volume* is sampled, these endmember contributions reflect *volume* contributions to the sampled soil volume defined by the sampling depth over a given area (all calculations and code shared here use 1 hectare (ha) by default). The endmember contributions reflect three unknowns. Hence, we set up two additional equations reflecting mass conservation of immobile elements as well as mobile base cations respectively.

$$[j]_s X_s \rho_s + [j]_f X_f \rho_f + [j]_{wf} X_{wf} \rho_{wf} = [j]_{mix,t=n}(X_s \rho_s + X_f \rho_f + X_{wf} \rho_{wf}) \quad \text{S4}$$

$$[i]_s X_s \rho_s + [i]_f X_f \rho_f + [i]_{wf} X_{wf} \rho_{wf} = [i]_{mix,t=n}(X_s \rho_s + X_f \rho_f + X_{wf} \rho_{wf}) \quad \text{S5}$$

S1.1.1 Simplified weathered feedstock endmember case

We first develop the mass balance framework using simplified assumptions for the weathered feedstock endmember:

$$[j]_{wf} = [j]_s \text{ , and} \quad \text{S6a}$$

$$\rho_{wf} = \rho_s \quad \text{S6b}$$

Substituting S6a and S6b into S4 and S5:

$$[j]_s X_s \rho_s + [j]_f X_f \rho_f + [j]_s X_{wf} \rho_s = [j]_{mix,t=n}(X_s \rho_s + X_f \rho_f + X_{wf} \rho_s) \quad \text{S7}$$

$$[i]_s X_s \rho_s + [i]_f X_f \rho_f + [i]_{wf} X_{wf} \rho_s = [i]_{mix,t=n}(X_s \rho_s + X_f \rho_f + X_{wf} \rho_s) \quad \text{S8}$$

Now, rearranging eq. S7 and S8 to isolate the endmember contributions on one side of the equation:

$$X_s \rho_s ([j]_{mix,t=n} - [j]_s) + X_f \rho_f ([j]_{mix,t=n} - [j]_f) + X_{wf} \rho_s ([j]_{mix,t=n} - [j]_s) = 0 \quad \text{S9}$$

$$X_s \rho_s ([i]_{mix,t=n} - [i]_s) + X_f \rho_f ([i]_{mix,t=n} - [i]_f) + X_{wf} \rho_s ([i]_{mix,t=n} - [i]_{wf}) = 0 \quad \text{S10}$$

Next, we substitute *$[i]_{wf}$* from eq. S2 into S10:

$$X_s \rho_s ([i]_{mix,t=n} - [i]_s) + X_f \rho_f ([i]_{mix,t=n} - [i]_f) + X_{wf} \rho_s \left([i]_{mix,t=n} - [i]_s - {}^{\rho_f}/_{\rho_s} [i]_f\right) = 0 \quad \text{S11}$$

For clarity, we rewrite the system of equations (S3, S7, S8) in matrix form:

$$\begin{pmatrix} \rho_s([j]_{mix,t=n} - [j]_s) & \rho_f([j]_{mix,t=n} - [j]_f) & \rho_s([j]_{mix,t=n} - [j]_s) \\ \rho_s([i]_{mix,t=n} - [i]_s) & \rho_f([i]_{mix,t=n} - [i]_f) & \rho_s\left([i]_{mix,t=n} - [i]_s - {}^{\rho_f}/_{\rho_s} [i]_f\right) \\ 1 & 1 & 1 \end{pmatrix} * \begin{pmatrix} X_s \\ X_f \\ X_{wf} \end{pmatrix} = \begin{pmatrix} 0 \\ 0 \\ 1 \end{pmatrix} \quad \text{S12}$$

For clarity, defining the following shorthand notions:

$$a \equiv \rho_s([j]_{mix,t=n} - [j]_s) = \rho_s \Delta[j]_s \quad \text{S13a}$$

$$b \equiv \rho_f([j]_{mix,t=n} - [j]_f) = \rho_f \Delta[j]_f \quad \text{S13b}$$

$$c \equiv \rho_s([i]_{mix,t=n} - [i]_s) = \rho_s \Delta[i]_s \quad \text{S13c}$$

$$d \equiv \rho_f([i]_{mix,t=n} - [i]_f) = \rho_f \Delta[i]_f \quad \text{S13d}$$

$$e \equiv \rho_f [i]_f \quad \text{S13e}$$

With these shorthand notations, equation S12 becomes:

$$\begin{pmatrix} a & b & a \\ c & d & c-e \\ 1 & 1 & 1 \end{pmatrix} * \begin{pmatrix} X_s \\ X_f \\ X_{wf} \end{pmatrix} = \begin{pmatrix} 0 \\ 0 \\ 1 \end{pmatrix} \quad \text{S14}$$

Now defining a new variable reflecting the sum of the soil and weathered feedstock residue endmembers:

$$X'_s = X_s + X_{wf} \quad \text{S15}$$

Inserting S15 into S14, the system of equations reduces to:

$$aX'_s + bX_f = 0 \quad \text{S16}$$

$$cX'_s + dX_f - eX_{wf} = 0 \quad \text{S17}$$

$$X'_s + X_f = 1 \quad \text{S18}$$

Now we solve for X'_s by substituting $X_f = 1 - X'_s$ from equation S18 into S16:

$$aX'_s + b(1 - X'_s) = 0 \quad \text{S19a}$$

$$aX'_s + b - bX'_s = 0 \quad \text{S19b}$$

$$(a-b)X'_s + b = 0 \quad \text{S19c}$$

$$X'_s = \frac{-b}{a-b} \quad \text{S19d}$$

Now, substituting, S19d into $X_f = 1 - X'_s$ from equation S18:

$$X_f = 1 - \frac{-b}{a-b} = \frac{a-b}{a-b} - \frac{-b}{a-b} = \frac{a-b+b}{a-b} = \frac{a}{a-b} \quad \text{S20}$$

Substituting S13a and S13b into S20:

$$X_f = \frac{\rho_s([j]_{mix,t=n} - [j]_s)}{\left(\rho_s([j]_{mix,t=n} - [j]_s) - \rho_f([j]_{mix,t=n} - [j]_f)\right)} = \frac{\rho_s \Delta[j]_s}{\rho_s \Delta[j]_s - \rho_f \Delta[j]_f} \quad \text{S21}$$

Now we substitute S20 and S19d into S17 to solve for X_{wf}:

$$c\frac{-b}{a-b}+d\frac{a}{a-b}-eX_{wf}=0 \quad \text{S22}$$

$$\frac{-bc+ad}{a-b}-eX_{wf}=0 \quad \text{S23}$$

$$X_{wf}=\frac{ad-bc}{e(a-b)} \quad \text{S24}$$

Finally, substituting S13a-S13e into S24:

$$X_{wf}=\frac{\rho_s\Delta[j]_s\rho_f\Delta[i]_f-\rho_f\Delta[j]_f\rho_s\Delta[i]_s}{\rho_f[i]_f(\rho_s\Delta[j]_s-\rho_f\Delta[j]_f)}=\frac{\rho_s\rho_f(\Delta[j]_s\Delta[i]_f-\Delta[j]_f\Delta[i]_s)}{\rho_f[i]_f(\rho_s\Delta[j]_s-\rho_f\Delta[j]_f)}=\frac{\rho_s(\Delta[j]_s\Delta[i]_f-\Delta[j]_f\Delta[i]_s)}{[i]_f(\rho_s\Delta[j]_s-\rho_f\Delta[j]_f)} \quad \text{S25}$$

Now, we first derive the equation for the mass transfer coefficient or dissolution factor, τ_j. In a given mixture, within the three-endmember mixing framework, the fraction of soil stays constant through time. The initial fraction of feedstock X_F is everything that is not soil. Over time, this fraction is split into a fraction that has weathered (X_{wf}) and one that has not yet (X_f).

$$X_F\equiv X_f+X_{wf}=1-X_s \quad \text{S26}$$

The mass transfer coefficient is the factor that splits X_F into X_{wf} and X_f:

$$X_{wf}=\tau X_F \quad \text{S27a}$$

$$X_f=(1-\tau)X_F \quad \text{S27b}$$

Solving S27a for τ and substituting S26:

$$\tau_j=\frac{X_{wf}}{X_F}=\frac{X_{wf}}{X_f+X_{wf}}\left(=\frac{X_{wf}}{1-X_s}\right) \quad \text{S28}$$

Finally, substituting S21 and S25 into S28, and then simplifying by canceling the same denominator $(\rho_s \Delta[j]_s - \rho_f \Delta[j]_f)$ as well as ρ_s:

$$\tau_j = \frac{\frac{\rho_s(\Delta[j]_s\Delta[i]_f - \Delta[j]_f\Delta[i]_s)}{[i]_f(\rho_s\Delta[j]_s - \rho_f\Delta[j]_f)}}{\frac{\rho_s\Delta[j]_s}{\rho_s\Delta[j]_s - \rho_f\Delta[j]_f} + \frac{\rho_s(\Delta[j]_s\Delta[i]_f - \Delta[j]_f\Delta[i]_s)}{[i]_f(\rho_s\Delta[j]_s - \rho_f\Delta[j]_f)}} = \frac{\rho_s/[i]_f(\Delta[j]_s\Delta[i]_f - \Delta[j]_f\Delta[i]_s)}{\rho_s\Delta[j]_s + \rho_s/[i]_f(\Delta[j]_s\Delta[i]_f - \Delta[j]_f\Delta[i]_s)} =$$

$$\frac{1/[i]_f(\Delta[j]_s\Delta[i]_f - \Delta[j]_f\Delta[i]_s)}{\Delta[j]_s + 1/[i]_f(\Delta[j]_s\Delta[i]_f - \Delta[j]_f\Delta[i]_s)} \quad \text{S29}$$

Now, multiplying with $[i]_f$:

$$\tau_j = \frac{\Delta[j]_s\Delta[i]_f - \Delta[j]_f\Delta[i]_s}{\Delta[j]_s[i]_f + \Delta[j]_s\Delta[i]_f - \Delta[j]_f\Delta[i]_s} \quad \text{S30}$$

Note that all densities cancel out, and τ_j is invariant to any density assumptions in the simplified case.

S1.1.2 Non-simplified weathered feedstock endmember case

We now also derive the framework for the non-simplified case where the weathered feedstock composition can be assigned independently. This may be particularly useful when constraints on such a composition exist, e.g. from natural analogues of soils developed on the feedstock regolith.

In this case we do not substitute S6a and S6b into S4 and S5, but nevertheless isolate the endmember contributions.

$$X_s\rho_s([j]_{mix,t=n} - [j]_s) + X_f\rho_f([j]_{mix,t=n} - [j]_f) + X_{wf}\rho_{wf}([j]_{mix,t=n} - [j]_{wf}) = 0 \quad \text{S31}$$

$$X_s\rho_s([i]_{mix,t=n} - [i]_s) + X_f\rho_f([i]_{mix,t=n} - [i]_f) + X_{wf}\rho_{wf}([i]_{mix,t=n} - [i]_{wf}) = 0 \quad \text{S32}$$

With

$$a_1 \equiv \rho_s([j]_{mix,t=n} - [j]_s) = \rho_s\Delta[j]_s \quad \text{S33a}$$

$$b_1 \equiv \rho_f([j]_{mix,t=n} - [j]_f) = \rho_f \Delta[j]_f \quad \text{S33b}$$

$$c_1 \equiv \rho_{wf}([j]_{mix,t=n} - [j]_{wf}) = \rho_{wf} \Delta[j]_{wf} \quad \text{S33c}$$

$$a_2 \equiv \rho_s([i]_{mix,t=n} - [i]_s) = \rho_s \Delta[i]_s \quad \text{S33d}$$

$$b_2 \equiv \rho_f([i]_{mix,t=n} - [i]_f) = \rho_f \Delta[i]_f \quad \text{S33e}$$

$$c_2 \equiv \rho_{wf}([i]_{mix,t=n} - [i]_{wf}) = \rho_{wf} \Delta[i]_{wf} \quad \text{S33f}$$

We do not substitute S2 into c_2 for this derivation, though in practice this is how users may wish to constrain the immobile element composition of the weathered feedstock endmember.

Now, substituting S33a to c into S31 and S33d to f into S32:

$$a_1 X_s + b_1 X_f + c_1 X_{wf} = 0 \quad \text{S34}$$

$$a_2 X_s + b_2 X_f + c_2 X_{wf} = 0 \quad \text{S35}$$

Solve S34 for X_s

$$X_s = \frac{-b_1}{a_1} X_f + \frac{-c_1}{a_1} X_{wf} \quad \text{S36}$$

Substitute S36 into S35, solve for X_f:

$$a_2\left(\frac{-b_1}{a_1} X_f + \frac{-c_1}{a_1} X_{wf}\right) + b_2 X_f + c_2 X_{wf} = 0$$

$$\frac{-b_1 a_2}{a_1} X_f + \frac{-c_1 a_2}{a_1} X_{wf} + b_2 X_f + c_2 X_{wf} = 0$$

$$\left(b_2 - \frac{b_1 a_2}{a_1}\right) X_f + \left(c_2 - \frac{c_1 a_2}{a_1}\right) X_{wf} = 0$$

$$X_f = -\frac{\left(c_2 - \frac{c_1 a_2}{a_1}\right)}{\left(b_2 - \frac{b_1 a_2}{a_1}\right)} X_{wf} \quad \text{S37}$$

Define D:

$$D \equiv -\frac{c_2 - \frac{c_1 a_2}{a_1}}{b_2 - \frac{b_1 a_2}{a_1}} \quad \text{S38}$$

Substitute S38 into S37:

$$X_f = D X_{wf} \quad \text{S39}$$

Now substitute S39 into S36:

$$X_s = \frac{-b_1 D}{a_1} X_{wf} + \frac{-c_1}{a_1} X_{wf} = \left(\frac{-b_1 D}{a_1} + \frac{-c_1}{a_1}\right) X_{wf} \quad \text{S40}$$

Define E:

$$E \equiv \frac{-b_1 D - c_1}{a_1} \quad \text{S41}$$

Substitute S41 into S40

$$X_s = E X_{wf} \quad \text{S42}$$

Substitute S39 and S42 into S3 and solve for X_{wf}

$$E X_{wf} + D X_{wf} + X_{wf} = 1$$

$$(E + D + 1) X_{wf} = 1$$

$$X_{wf} = \frac{1}{(E + D + 1)} \quad \text{S43}$$

Substitute S43 into S39 and S42:

$$X_f = \frac{D}{(E + D + 1)} \quad \text{S44}$$

$$X_s = \frac{E}{(E + D + 1)} \quad \text{S45}$$

Now, substitute S43 and S44 into the τ_j expression S28:

$$\tau_j = \frac{\frac{1}{(E+D+1)}}{\frac{1}{(E+D+1)}+\frac{D}{(E+D+1)}} = \frac{1}{1+D} \quad \text{S46}$$

Now we further simplify D for the calculation of τ.

$$D \equiv -\frac{c_2-\frac{c_1 a_2}{a_1}}{b_2-\frac{b_1 a_2}{a_1}} \quad \text{S38}$$

Substituting equations S33a to S33d into $\frac{c_1 a_2}{a_1}$ and $\frac{b_1 a_2}{a_1}$:

$$\frac{c_1 a_2}{a_1} = \frac{\rho_{wf}\Delta[j]_{wf}\rho_s\Delta[i]_s}{\rho_s\Delta[j]_s} = \rho_{wf}\frac{\Delta[j]_{wf}\Delta[i]_s}{\Delta[j]_s} \quad \text{S47}$$

$$\frac{b_1 a_2}{a_1} = \frac{\rho_f\Delta[j]_f\rho_s\Delta[i]_s}{\rho_s\Delta[j]_s} = \rho_f\frac{\Delta[j]_f\Delta[i]_s}{\Delta[j]_s} \quad \text{S48}$$

Now, substituting S47 and S48 as well as S33f and S33e into S38:

$$D \equiv -\frac{c_2-\frac{c_1 a_2}{a_1}}{b_2-\frac{b_1 a_2}{a_1}} = -\frac{\rho_{wf}\Delta[i]_{wf}-\rho_{wf}\frac{\Delta[j]_{wf}\Delta[i]_s}{\Delta[j]_s}}{\rho_f\Delta[i]_f-\rho_f\frac{\Delta[j]_f\Delta[i]_s}{\Delta[j]_s}} = -\frac{\rho_{wf}}{\rho_f}\frac{\Delta[i]_{wf}-\frac{\Delta[j]_{wf}\Delta[i]_s}{\Delta[j]_s}}{\Delta[i]_f-\frac{\Delta[j]_f\Delta[i]_s}{\Delta[j]_s}} \quad \text{S49}$$

Substituting S49 into S46:

$$\tau = \frac{1}{1-\frac{\rho_{wf}}{\rho_f}\frac{\Delta[i]_{wf}-\frac{\Delta[j]_{wf}\Delta[i]_s}{\Delta[j]_s}}{\Delta[i]_f-\frac{\Delta[j]_f\Delta[i]_s}{\Delta[j]_s}}} \quad \text{S50}$$

While soil density cancels out also for the non-simplified case, this is not true for the densities of the weathered feedstock and feedstock endmembers. The potential bias introduced if these terms are quantified or estimated erroneously is assessed in the sensitivity analysis section.

Note also that in this scenario, as $\rho_{wf} \neq \rho_s$, in the absence of empirical data $[i]_{wf}$ needs to be estimated differently to S2 in the simplified case:

$$[i]_{wf} = \frac{\rho_s v_{sampled\ layer}\,[i]_s + \rho_f v_{sampled\ layer}[i]_f}{\rho_{wf} v_{sampled\ layer}} = \frac{\rho_s[i]_s + \rho_f[i]_f}{\rho_{wf}} = {}^{\rho_s}/_{\rho_{wf}}\,[i]_s + {}^{\rho_f}/_{\rho_{wf}}\,[i]_f \quad \text{S51}$$

S1.1.3 Adjustment of CDR potential in non-simplified SOMBA framework

The analysis presented here assumes that mobile base cations are transported out of the sampled layer following feedstock weathering, although in reality some degree of cation retention within the soil matrix is expected, particularly over the short to intermediate timescales relevant for enhanced weathering (EW). This retention can occur for example if a fraction of the feedstock does not weather or through incorporation into secondary phases such as clays and oxides. Such processes are well documented in natural weathering environments and must be considered when interpreting dissolution-based carbon dioxide removal (CDR) estimates.

Both the simplified and non-simplified SOMBA formulations are capable of representing this behavior, albeit in different but physically consistent ways. In the simplified framework, which assumes that the weathered feedstock mobile element composition converges toward that of the background soil, cation retention manifests naturally as an apparent limit to weathering progress: post-weathering compositions never reach the base of the mixing diagram because not all cations are lost, and τ_j never reaches 1. This behavior yields accurate and conservative CDR estimates relative to the cations that were actually mobilized, without implying carbon removal where none occurred. From a verification perspective, this is advantageous, as it ensures that undissolved or retained cations are not inadvertently counted toward realized CDR.

In the non-simplified framework, the composition of the weathered-feedstock endmember is explicitly adjusted to reflect partial retention of base cations. Here, τ_j can physically reach 100% because the retained fraction is incorporated into the endmember composition, effectively redefining the “completely weathered” state. Importantly, this approach requires that the gross CDR potential of the feedstock be reduced by the same fraction of retained cations:

$$CDR_{adjusted} = CDR_{pot} * \left(1 - f_{j,retained}\right) \approx CDR_{pot} \frac{[j]_f - [j]_{wf}}{[j]_f - [j]_s} \quad \text{S51}$$

Without such an adjustment, the framework would overestimate total CDR by counting both the released and the retained cations as weathered. This highlights that the difference between the simplified and non-simplified approaches is primarily one of accounting convention: the simplified approach reports 100% relative to the total feedstock CDR potential but never reaches it in practice, whereas the non-simplified formulation normalizes to the subset of cations that are actually mobile, reducing total realizable CDR potential—but where 100% weathering can actually be achieved.

We demonstrate this effect using a hypothetical case in which 20% of base cations are retained within the weathered feedstock due to the presence of a residual, non-weathering phase. For simplicity, we neglect mineralogical controls and assume that this residual phase constitutes 20% of the initial feedstock volume, containing 20% of both mobile and immobile elements. Under these conditions, the composition of the weathered-feedstock endmember can be estimated by combining 20% of the original feedstock composition with 80% of the background soil that replaces the lost feedstock volume within a layer equivalent to the sampling depth. The immobile elements originally contained in the weathered fraction of the feedstock are assumed to remain within the sampled layer, consistent with the SOMBA mass-balance formulation. The mobile and immobile element composition of this weathered feedstock composition can then be estimated as:

$$[j]_{wf} = \frac{0.8\rho_s v_{sampled\ layer}\,[j]_s + 0.2\rho_f v_{sampled\ layer}[j]_f}{0.2\rho_f v_{sampled\ layer} + 0.8\rho_s v_{sampled\ layer}} \quad \text{S52}$$

$$[i]_{wf} = \frac{0.8\rho_s v_{sampled\ layer}\,[i]_s + \rho_f v_{sampled\ layer}[i]_f}{0.2\rho_f v_{sampled\ layer} + 0.8\rho_s v_{sampled\ layer}} \quad \text{S53}$$

$$[\rho]_{wf} = 0.2\rho_f + 0.8\rho_s \quad \text{S54}$$

When available, the estimation of the composition of the weathered feedstock endmember could also be informed by mineralogical data, e.g., by assuming that accessory minerals with low weathering rates are not going to resolve on relevant timelines. For demonstration purposes, we assume that 1500 t ha^{-1} of such a feedstock are applied (large value purely chosen for visibility in Figure S1; τ_j analysis invariant to application), and that 100% of this feedstock has weathered. The

composition of this post-weathering composition (as calculated using the non-simplified case) is shown in Figure S1a and b.

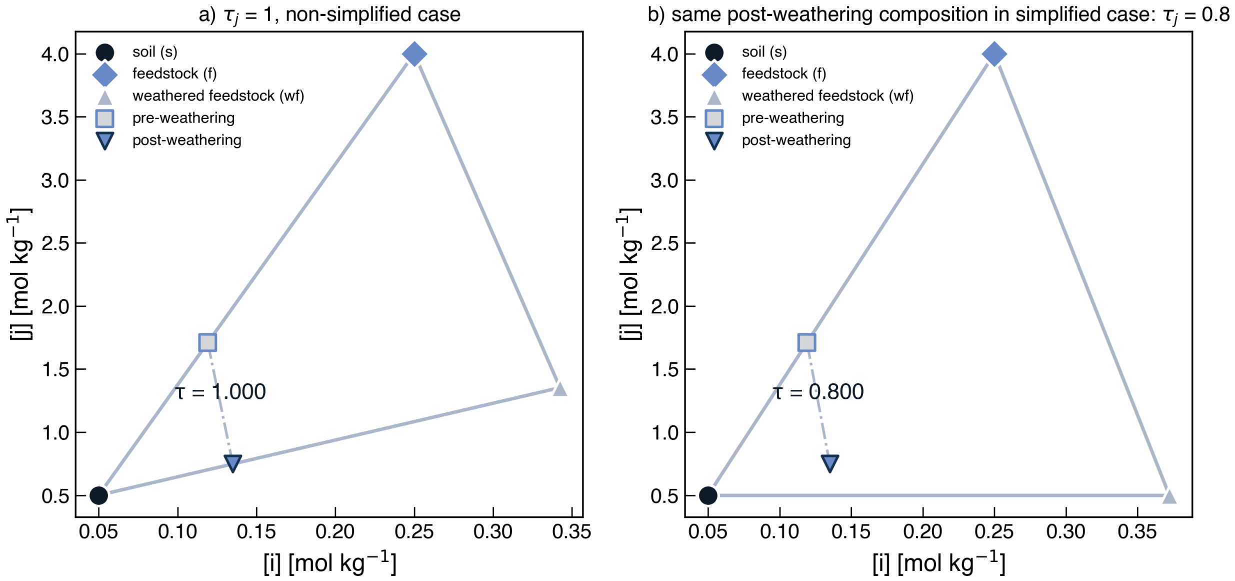

Figure S1: Demonstration of the difference in the non-simplified and simplified case when constraining τ_j based on weathered feedstock compositions representing non-complete weathering.

When the non-simplified SOMBA framework is applied to this post-weathering composition (SOMBA_tau), the calculated dissolution fraction (τ_j) is correctly recovered as 1.0, consistent with the position of the mixture on the lower boundary of the model domain. In contrast, when the simplified framework (SOMBA_tau_simplified) is applied to the same post-weathering composition, the estimated τ_j value is 0.8 (Figure S1b), reflecting its position above the lower boundary due to the reduced mobile element concentration of the weathered feedstock endmember. This apparent τ_j value of 0.8 corresponds precisely to the 80% of cations that were actually released from the feedstock, with the remaining 20% retained in the residual phase. Thus, both formulations yield consistent and physically meaningful results: the simplified framework indicates that 80% of the total feedstock CDR potential has been realized, whereas the non-simplified framework indicates complete weathering of the feedstock fraction capable of contributing to CDR, after accounting for cation retention.

In both cases, SOMBA provides a physically consistent and conservative representation of realized weathering, ensuring that calculated dissolution fractions and associated CDR reflect measurable, leaching-based carbon drawdown. Whether one elects to normalize to the original feedstock composition or to a corrected "effective" feedstock potential depends on the intended application: for crediting and MRV purposes, the simplified approach offers a built-in safeguard against overestimation (and is also invariant to system densities), while for research and mechanistic studies, the non-simplified formulation may be preferable as it allows more explicit treatment of mineral-specific dissolution and cation retention pathways.

Finally, we emphasize that the sensitivity analyses conducted here demonstrate that modest degrees of cation retention introduce only minor biases to τ_j under both frameworks. Across a wide range of plausible retention scenarios ($[j]_{wf}/[j]_s$ = 0.75–1.25), the resulting deviations in τ_j remain below ~20% (see section 3.3). These findings confirm that the SOMBA framework is not only theoretically robust but also empirically stable under realistic field conditions.

S1.2 Calculation of deployment parameters from post-weathering samples

From the estimates of endmember contributions to the post-weathering soil-feedstock mix sample as well as the rock powder dissolution calculated using the approach outlined above, additional deployment parameters can be calculated that may be valuable for the purposes of MRV. First, we can calculate the mass of rock powder initially added to the sampled soil volume (a, t ha^{-1}):

$$a = fV_{f,t=0}\, v_{sampled\ layer}\, \rho_f \qquad \text{S55}$$

where $fV_{f,t=0}$ is the pre-weathering feedstock volume fraction, defined as the sum of the volume fraction comprising residual feedstock as well as the initial feedstock present that has since weathered:

$$fV_{f,t=0} = X_f + X_{wf} \qquad \text{S56}$$

and the sampled layer volume per hectare is calculated from the sampling depth ($d_{sampling}$):

$$v_{sampled\ layer}[m^3\ ha^{-1}] = 10000\ m^2 ha^{-1} * d_{sampling}[m] \quad \text{S57}$$

Note that in cases where the sampling depth is not the same as the soil mixing depth, it is important to use the depth of soil sampling. If the two depths are not the same, using calculated parameters that are based only on the sampled layer for the entire mixed layer assumes that the sampled layer composition is representative of the entire mixed layer. This is not necessarily the case, particularly when feedstock distribution is not uniform with depth. In cases where the mixing depth is larger than the sampling depth, one could assume that the calculated dissolution fraction (τ_j) applies to the known application amount, but this would trade off against the benefit that feedstock addition can be calculated from the enrichment of immobile elements. To make sure that the calculation of initial CDR from a combination of τ_j with the applied feedstock mass is entirely constrained in empirical measurements the choice of sampling depth should ideally be equal to the mixing depth.

In addition, we can calculate the initial soil-feedstock mix composition from the post-weathering composition and mixing model outputs. Initial concentrations can be calculated by combining the amounts of base cations as well as immobile elements contributed to the initial mix from both soil and feedstock divided by the mass of the system:

$$[j]_{mix,\ t=0} = \frac{\rho_s\, v_{s,t=0}\, [j]_s + \rho_f\, v_{f,t=0}\, [j]_f}{\rho_s\, v_{s,t=0} + \rho_f\, v_{f,t=0}} \quad \text{S58}$$

$$[i]_{mix,\ t=0} = \frac{\rho_s\, v_{s,t=0} [i]_s + \rho_f\, v_{f,t=0}\, [i]_f}{\rho_s\, v_{s,t=0} + \rho_f\, v_{f,t=0}} \quad \text{S59}$$

where $v_{s,t=0}$ and $v_{f,t=0}$ are the area normalized volumes ($m^3\ ha^{-1}$) of soil and feedstock within the sampled topsoil volume:

$$v_{s,t=0} = v_{sampled\ layer}\, X_s \quad \text{S60}$$

$$v_{f,t=0} = v_{sampled\ layer}\, fV_{f,t=0} = v_{sampled\ layer}(X_f + X_{wf}) \quad \text{S61}$$

Substituting S60 and S61 in S58 and S59:

$$[j]_{mix,\,t=0} = \frac{\rho_s\, v_{sampled\ layer}\, X_s\, [j]_s + \rho_f\, v_{sampled\ layer} (X_f + X_{wf}) [j]_f}{\rho_s\, v_{sampled\ layer}\, X_s + \rho_f\, v_{sampled\ layer} (X_f + X_{wf})} = \frac{\rho_s\, X_s\, [j]_s + \rho_f (X_f + X_{wf}) [j]_f}{\rho_s\, X_s + \rho_f (X_f + X_{wf})} \quad \text{S62}$$

$$[i]_{mix,t=0} = \frac{\rho_s v_{sampled\ layer}\, X_s\, [i]_s + \rho_f\, v_{sampled\ layer} (X_f + X_{wf}) [i]_f}{\rho_s\, v_{sampled\ layer}\, X_s + \rho_f v_{sampled\ layer} (X_f + X_{wf})} = \frac{\rho_s\, X_s\, [i]_s + \rho_f (X_f + X_{wf}) [i]_f}{\rho_s\, X_s + \rho_f (X_f + X_{wf})} \quad \text{S63}$$

The calculation of feedstock application mass as well as pre-weathering composition from post-weathering composition and deployment data is included in the SOMBA_tau_meta_simplified and SOMBA_tau_meta functions defined in the SOMBA.py file in the supplement.

S1.3 Pre-weathering mix composition

After addition of rock powder to soils, the composition of the soil-rock-powder mix falls on a mixing line between both endmembers (**Error! Reference source not found.**a). Provided the rock powder is enriched in both base cations as well as at least one immobile element compared to the baseline soil, the addition of rock powder causes an increase of both base cation and immobile element concentrations. The pre-weathering mix concentrations of both major cations (j) and an immobile element (i; both in mol/kg) can be calculated from the mix of both endmembers:

$$[j]_{mix,t=0} = j_f\, r_{m,t=0} + j_s\, (1 - fM_{f,t=0}) \quad \text{S64}$$

$$[i]_{mix,t=0} = i_f\, r_{m,t=0} + i_s\, (1 - fM_{f,t=0}) \quad \text{S65}$$

Where the subscripts *f* and *s* denote feedstock and soil respectively, and fM_f refers to the mass mixing ratio of feedstock in the soil-feedstock mix, which can be calculated as:

$$fM_{f,t=0} = \frac{a}{(a + m_{soil,t=0})} \quad \text{S66}$$

where *a* is the application amount of rock powder (in t ha^{-1}), and $m_{topsoil,t=0}$ is the mass of soil in the mixed soil rock powder topsoil layer right after deployment (in t ha^{-1}). The application amount *a* is in this case given from deployment data, while the mass of topsoil can be calculated from the topsoil volume that is not rock powder (units in square brackets):

$$m_{soil,t=0}\ [t\ ha^{-1}] = v_{soil,t=0}[m^3 ha^{-1}]\ \rho_{soil}[t\ m^{-3}] \quad \text{S67}$$

$$v_{soil,t=0}[m^3 ha^{-1}] = v_{mixed\ layer}[m^3 ha^{-1}] - v_f[m^3 ha^{-1}] \quad \text{S68}$$

$$v_{f,t=0}[m^3 ha^{-1}] = \frac{a\ [t\ ha^{-1}]}{\rho_f\ [t\ m^3]} \quad \text{S69}$$

$$v_{mixed\ layer}[m^3 ha^{-1}] = 10000\ [m^2 ha^{-1}]\ d_{mix}\ [m] \quad \text{S70}$$

where *m* refers to mass, *v* to volume, and *ρ* to density of the soil within the mixed layer (subscript topsoil), the total mixed layer (mixed layer) as defined by the mixing depth (d_{mix}), as well as the feedstock (f). Substituting S67-S70 into S66:

$$fM_{f,t=0} = \frac{a}{(a+(10000\ d_{mix} - {}^{a}/_{\rho_f})\ \rho_{soil})} \quad \text{S71}$$

S1.4 Post-weathering composition

As feedstock dissolves, both base cation as well as immobile element concentrations change. While base cation concentrations decrease as these mobile elements are leached from topsoils, immobile element concentrations increase due to the loss of feedstock mass (and volume) from topsoils, resulting in a vector originating at the pre-weathering composition towards the bottom right in *j* vs. *i* space (**Error! Reference source not found.**a). For the simplified framework, the post-weathering soil-rock powder mix composition can be calculated as a function of feedstock dissolution (mass transfer coefficient τ_j) fraction through system mass conservation where the denominate describes the mass of the post-weathering mix and the numerator its amount base cations or immobile elements:

$$[j]_{mix,t=n} = \frac{\rho_s v_{s,t=n}[j]_s + \rho_f v_{f,t=n}[j]_f}{\rho_s v_{s,t=n} + \rho_f v_{f,t=n}} \quad \text{S72}$$

$$[i]_{mix,t=n} = \frac{\rho_s v_{s,t=n}[i]_s + \rho_f v_{f,t=0}[i]_f}{\rho_s v_{s,t=n} + \rho_f v_{f,t=n}} \quad \text{S73}$$

where the $t = 0$ in the numerator of eq. S73 reflects the fact that immobile elements added through feedstock are retained within topsoils upon weathering. Post-weathering soil and feedstock volumes can be calculated as:

$$v_{f,t=n} = v_{f,t=0}\,(1 - \tau_j) \quad \text{S74}$$

$$v_{s,t=n} = v_{f,t=0} + v_{f,t=0} - v_{f,t=n} = v_{s,t=0} + v_{f,t=0}\,\tau_j \quad \text{S75}$$

In the non-simplified case, the post-weathering composition can be calculated by mixing the three endmember contributions as follows:

S1.5 Internal consistency of the SOMBA framework

The code supplement (SOMBA_verification.py and SOMBA_verification_simplfied.py) contains code that demonstrates the internal consistency of the SOMBA framework for both the simplified and non-simplified frameworks. In the first part of the script, an example dataset is generated based on assumed deployment parameters. Some of these parameters—such as the amount of feedstock applied, the dissolution fraction, and others—are specifically required for the SOMBA_start, SOMBA_end, and SOMBA_end_simplified functions. These functions estimate the composition of the soil-feedstock mix before and after weathering, respectively, using deployment-specific inputs. However, when using the soil mass balance framework to estimate rock powder dissolution fractions from post-weathering samples, these parameters may not be necessary.

In the second part of the scripts, the generated datasets are used to sequentially call a series of soil mass balance functions defined in the SOMBA.py file, which are derived here. The functions called include: (1) SOMBA_start, which calculates the pre-weathering soil-feedstock mix composition from deployment parameters; (2) SOMBA_end or SOMBA_end_simplified, which estimate the post-weathering composition; (3) SOMBA_tau or SOMBA_tau_simplified, which calculate the rock powder dissolution fraction from deployment data, including baseline soil, feedstock, and post-weathering compositions; and (4) SOMBA_tau_meta or SOMBA_tau_meta_simplified , which perform the same calculation as SOMBA_tau and

SOMBA_tau_simplified but also provide metadata such as endmember contributions and detected feedstock amounts.

Finally, the exported Figure S2 demonstrates that the values calculated from the SOMBA framework (such as τ_j and pre-weathering concentrations) are equivalent to the values assumed or calculated *a-priori*. The same is true for the estimate rock powder application amount—in this case it is important to consider potential mismatches between mixing and sampling depth, where the detected rock powder amount is going to be less than the amount assumed *a-priori* if the sampling depth is less than the mixing depth. While Figure S2 shows the results for the simplified framework, the code SOMBA_verification.py exports the equivalent demonstration for the non-simplified case.

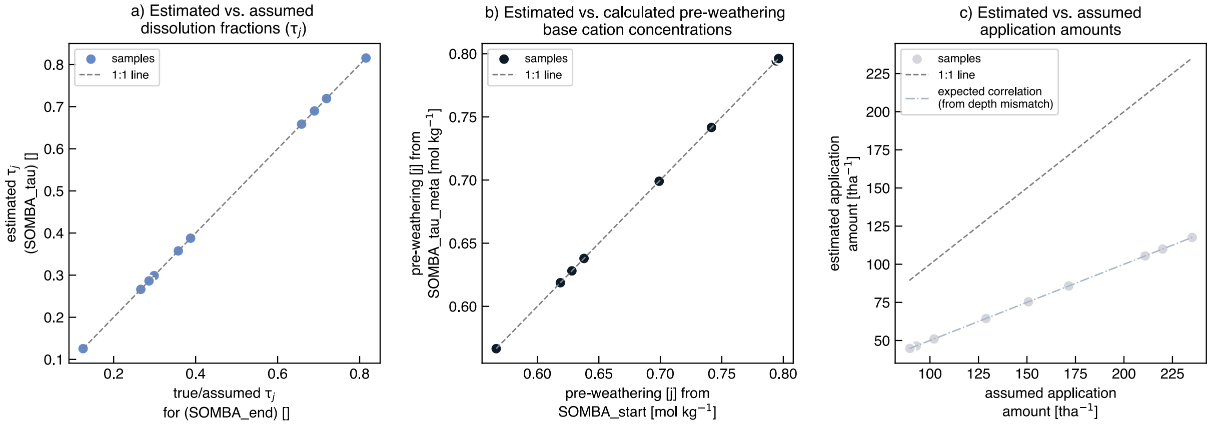

Figure S2: This figure demonstrates that the soil mass balance framework developed here is internally consistent for the simplified case. The calculated dissolution fraction τ_j (a) and pre-weathering soil concentration (b) are equivalent to the values assumed a-priori. The same is true for detected rock powder application amounts (c) when taking into account potential mismatches between soil mixing and sampling depth.

S2 Soil data

S2.1 Site and sampling information

Site 1: This field is classified as loamy-sand, with a mean pH of 7.0, and is on a corn-yellow pea rotation. The only fertilizer used is chicken litter.
Site 2: This field is classified as silt-loam, with a mean pH of 6.4. This field is used as pastureland, growing native grasses for grazing. There is no tillage, irrigation, fertilizer, or liming use.
Site 3: This field is classified as loamy-sand. This field is used for peanuts, and is irrigated by a center pivot. There is no tillage, regular nitrogen application, and highly infrequent liming.
Site 4 and 5: Sites 4 and 5 fields are managed by the same farmer. The fields are no-till and are ripped every 3 years. Both fields are on a corn-soy rotation, and receive nitrogen fertilizer during corn season. There is no irrigation. No pH data is available for this field.

Site 1, Site 2, and Site 3 fields were sampled in a grid array across the entire field region, with 2 cores taken at each sampling location and homogenized. Samples were dried at 60 °C, sieved to 2mm and ground prior to analysis. Site 4 and 5 samples were collected by randomly pooling 12 15-cm drill cores from a 1m radius circle. Samples were dried at 60 °C, sieved to 2mm and ground prior to analysis.

The locations of the field sites as well as of the soil samples used to constrain field composition are shown in Figure S3 below.

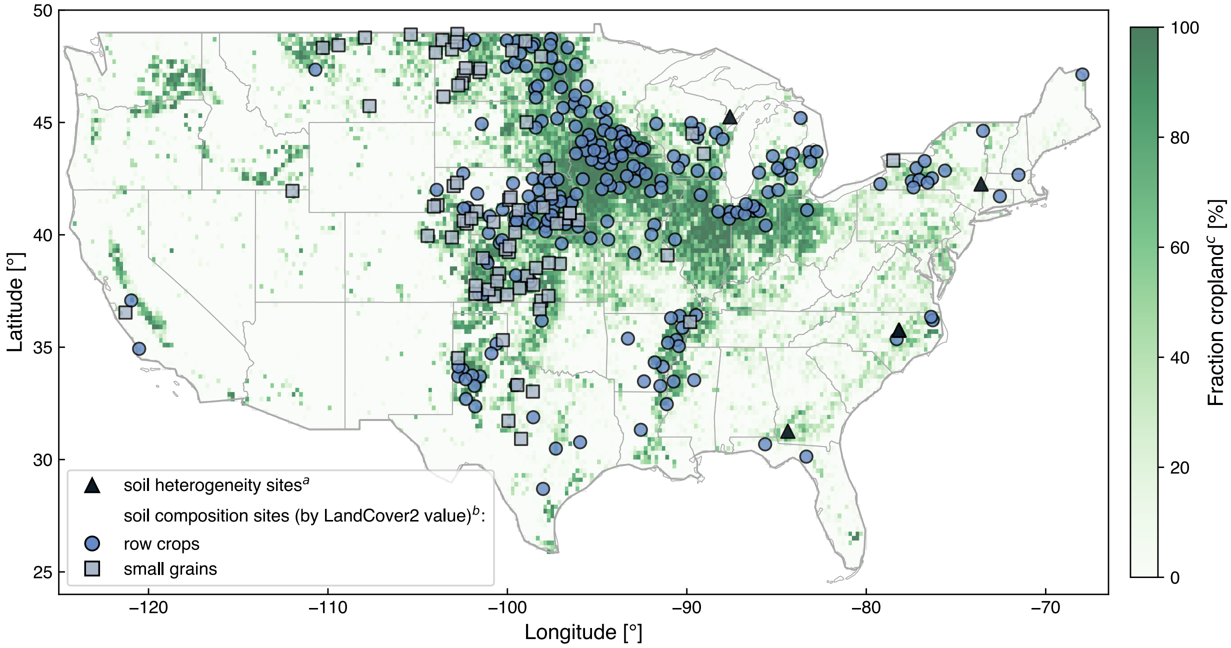

*Figure S3: Sites of the data utilized to constrain soil composition as well as in-field spatial heterogeneity (**Error! Reference source not found.**).*
[a] Novel soil heterogeneity dataset reported here.
b 1
c 2

S2.2 Analytical information

Powdered soil samples (typically 0.1 g) were leached in 12 ml of 1M Ammonium Acetate (trace metal grade) and centrifuged in 15 ml polypropylene tubes for 5 minutes at 4000 rpm to release any adsorbed cations (i.e, the exchangeable fraction) and subsequently washed in 2 ml of 2X MilliQ H_2O (18.2MΩcm at 25 °C) and centrifuged again. The soil was then transferred into pre-acid-cleaned quartz crucibles, dried at 60 °C and ashed at 600 °C to incinerate any organic matter (and release volatiles). The dried residue was weighed for insoluble content and to estimate the LOI. The residue was then transferred into pre-acid-cleaned teflon beakers and dissolved completely using a mixture of 5 ml distilled hydrochloric acid (HCl), 5 ml distilled nitric acid (HNO3) and 1 ml of trace metal grade hydrofluoric acid (HF), capped, heated at 100 °C for 24 hours. The samples were then uncapped and evaporated to dryness at 90 °C and redissolved in 5 ml of 6N HCl.

Splits were taken for elemental concentrations measurements. For analysis on the Agilent 8900 Triple Quadrupole ICP-MS, a split of 15 µl from each sample was evaporated, diluted 1000 times with 1% HNO3 (v/v) and spiked with ^{26}Mg and ^{49}Ti. Indium was introduced externally as an internal standard. For analysis on the Thermo Scientific Element XR ICP-MS, a split of 10 µl from each sample was evaporated, diluted 400 times with 5% HNO3 (v/v) and spiked with ^{26}Mg, ^{42}Ca, ^{49}Ti and 1ppb In. Values were normalized using routine measurements of USGS geostandards BHVO-2 and SGR-1b (processed with each batch of samples throughout the entire procedure), whose precision was within 1% of certified values (4% for Al) on the Agilent (LL). For more information on the analytical procedure see also Reershemius et al. (2023).

S2.3 Implementation of soil heterogeneity in Monte Carlo simulations

We use soil composition data from five novel field sites sampled at high spatial densities to constrain in-field heterogeneity for the Monte Carlo signal-to-noise analysis. The data are normalized by the field mean concentration (Figure S4) before we fit log-normal distributions to make sure the population means are 1. The use of log-normal (rather than normal) distributions is intentional because samples generated from log-normal distributions always have positive values, preventing the occurrence of non-physical negative soil concentrations in the signal-to-noise analysis without having to filter some data. For normal distributions, this could be achieved by simply filtering out negative model occurrences, but this would change the mean of generated sample distributions and cause a systematic error in calculated dissolution fractions. In addition, using log-normal compared to normal fits also represents a conservative choice for the signal-to-noise analysis due to the generally higher variance, as well has overall better fits compared to normal distributions (R^2 better for 12 out of 20 elemental field distributions).

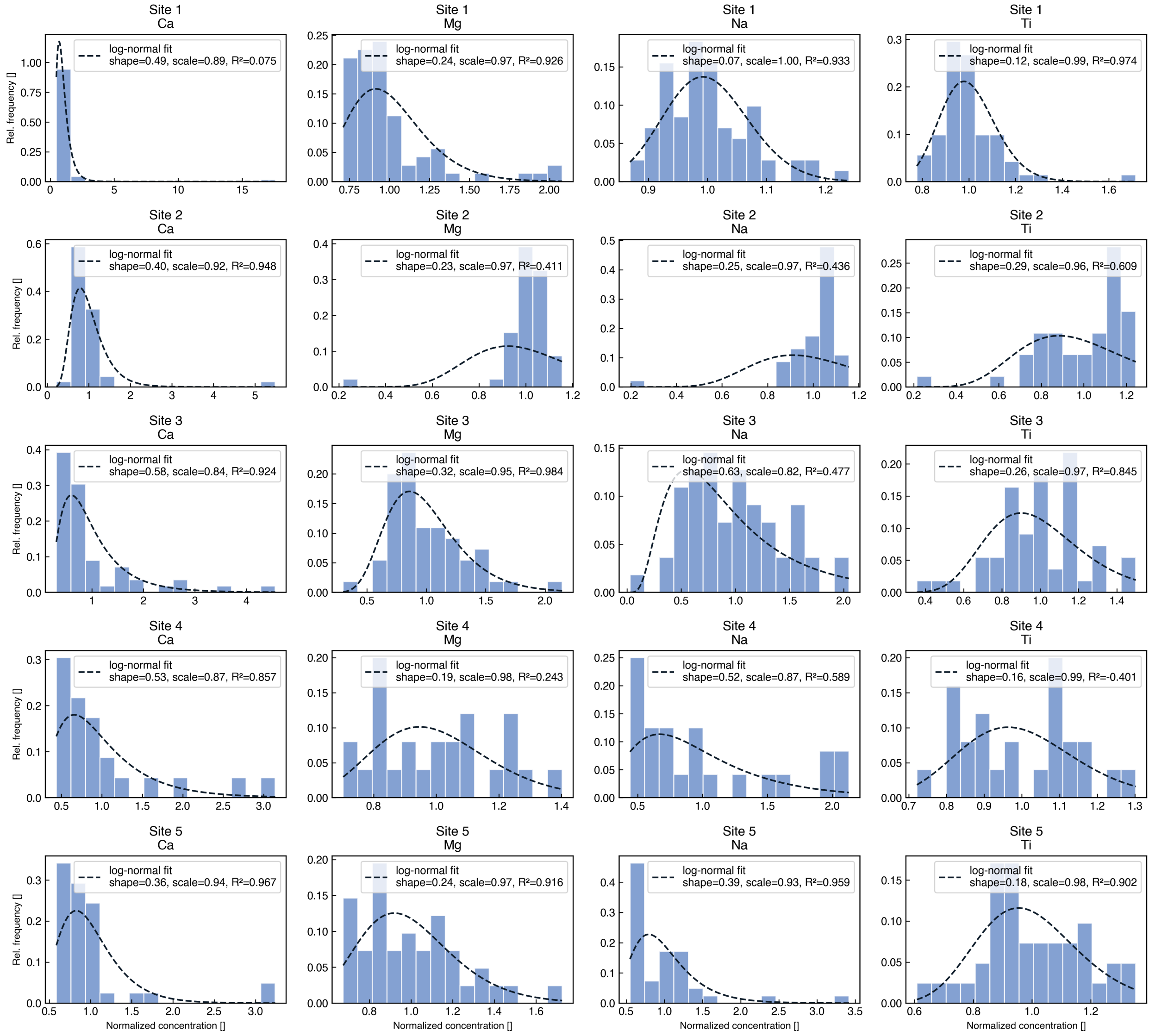

*Figure S4: Distributions of baseline data for the 5 field sites (**Error! Reference source not found.**) including log-normal fits to the data. The shape parameters, corresponding to the standard deviation of the normal distribution of the logarithm of the data, are plotted in Figure S5.*

Generally, a random variable is log-normally distributed if:

$$X \sim LogNormal(\mu, \sigma) \tag{S76}$$

Which means that:

$$ln(X) \sim N(\mu, \sigma^2) \tag{S77}$$

where μ is the mean, σ the standard deviation, and σ^2 the variance of the respective distributions, with log-normal distributions conventionally defined via the standard deviation of the underlaying normal distribution. The expected value (mean) of a log-normal variable X can be calculated as:

$$\mathrm{E[X]} = \mathrm{e}^{\left(\mu + \frac{\sigma^2}{2}\right)} \qquad \text{S78a}$$

Hence, when using the parameters of log-normal fits to populations with a given mean (Figure S4) to generate synthetic data for the Monte Carlo simulations, if generating μ and σ independently, the mean of the resulting populations will not be the same as of the initial distribution (i.e., 1). Or said differently, if we want the mean of a synthetic distribution to be a specific value, μ and σ are not independent—only one can be randomly generated. We implement this into the Monte Carlo simulation by randomly generating shape parameters (σ_{syn}) and then calculating μ_{syn} such that *E(X)* = 1:

$$\mathrm{E[X]} = \mathrm{e}^{\left(\mu_{syn} + \frac{\sigma_{syn}^2}{2}\right)} = 1 \qquad \text{S78b}$$

Now, taking the natural logarithm:

$$\ln\left(\mathrm{e}^{\left(\mu_{syn} + \frac{\sigma_{syn}^2}{2}\right)}\right) = \ln(1) \Rightarrow \mu_{syn} + \frac{\sigma_{syn}^2}{2} = 0 \Rightarrow \mu_{syn} = -\frac{\sigma_{syn}^2}{2} \qquad \text{S79}$$

The empirically constrained simulated μ_{syn} and σ_{syn} describe log-normal distributions with a mean of 1 and σ (shape) parameters constrained from field data (with a mean of 1), and are used to randomly generate sets of samples by multiplying these in-field variance factors with true “true” sample compositions.

Because the σ values from the fit to field data (Figure S4) are neither normally nor log-normally distributed (negative R^2; Figure S5), in the Monte Carlo simulations we generate synthetic σ_{syn} values by randomly pulling from uniform distributions set out by the minimum and maximum

observed σ values observed in field data (for Ca, Mg, and Na the used values are 0.072402 and 0.629872, and for Ti 0.119775 and 0.288003).

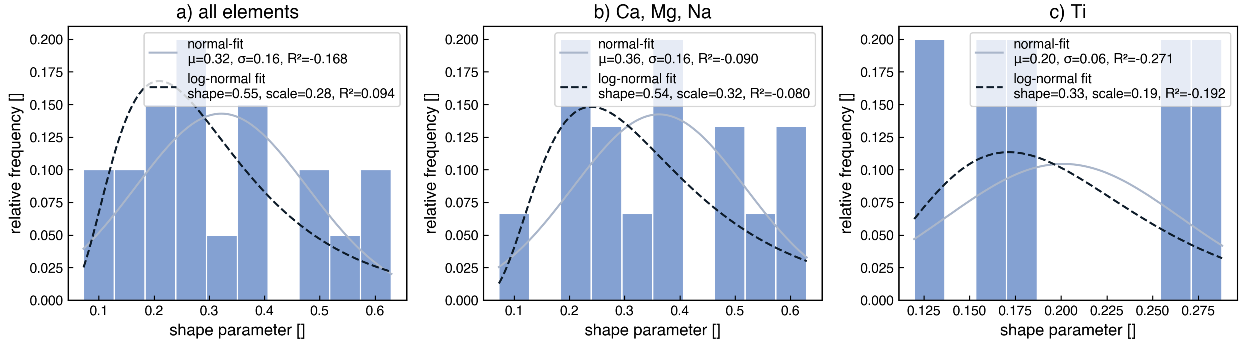

Figure S5: Histograms as well as normal and log-normal fits to the shape parameters from log-normal fits to soil data. The signal-to-noise analysis and related Monte Carlo simulations use uniform distribution set out by the minimum and maximum Ca, Mg, and Na shape values (b) as well as Ti shape values (c) due to low fit of both normal and log-normal distributions.

S3 Sensitivity analysis

S3.1 Sensitivity analysis of soil mass balance framework

S3.1.1 Immobile element enrichment

Accurately accounting for immobile element enrichment in soils due to feedstock loss from the topsoil system is essential to quantifying feedstock dissolution. Here, we demonstrate the impact of this process by simulating the increase in immobile element concentrations in post-weathering soil-feedstock mixtures for three different feedstock application amounts, the whole range of dissolution fractions, as well as soils with a Ti content that is 2-10 times depleted compared to US-average basalt.

As expected, the enrichment of immobile elements is highest at high dissolution fractions, τ_j, as well as at low feedstock:soil immobile element ratios (r_i), as shown in Figure S6**Error! Reference source not found.**a-c where the impact is visualized by showing the difference in post-weathering to pre-weathering soil-feedstock mix composition (Δi). At constant r_i, this effect is linear with increasing τ_j, demonstrating that it scales with the amount of feedstock volume that has been lost from the system and is replaced with soil when considering constant sampled topsoil volumes. There are also instances where this process will have a negligible (>5%) effect. The effect of immobile element enrichment due to mass loss also increases with increasing application amounts, because at the same dissolution fraction the volume of feedstock that is lost from topsoils increases. When r_i is defined via depletion relative to a fixed feedstock composition, Δi increases with decreasing r_i, reflecting the fact that when sampling constant soil volumes, soil that replaces lost feedstock has a higher immobile element concentration. Because r_i is defined as the ratio of feedstock to soil immobile element concentrations, lower r_i reflects higher soil concentrations of i:

$$[i]_s = \frac{[i]_f}{r_i} \qquad \text{S80}$$

Note that the results depend on mixing depth, with lower depth corresponding to a higher concentration of immobile elements; Figure S6 shows results for d_{mixing} = 0.2 m. There is also an

impact of feedstock dissolution on soil base cation concentrations beyond the pure loss of feedstock (section S3.2).

When this enrichment is not accounted for, the resulting dissolution fractions are overestimated. Here we demonstrate this effect by comparing the erroneously high τ_j* as calculated from equation 1 based on pre-weathering base cation concentrations estimated by vertically projecting post-weathering [i] concentrations onto the mixing line between soil and feedstock endmembers. The difference between the erroneous τ_j* and τ_j as calculated from the endmember approach is largest at intermediate τ_j and low r_i, where it goes up to a Δ value of 0.12 (Figure S6d-f). The relative effect is the highest at low τ_j and approaches 100% at low dissolution fractions and differences between soil and feedstock immobile element concentrations (Figure S6g-i). The impact on quantified dissolution fractions does not scale with application amount.

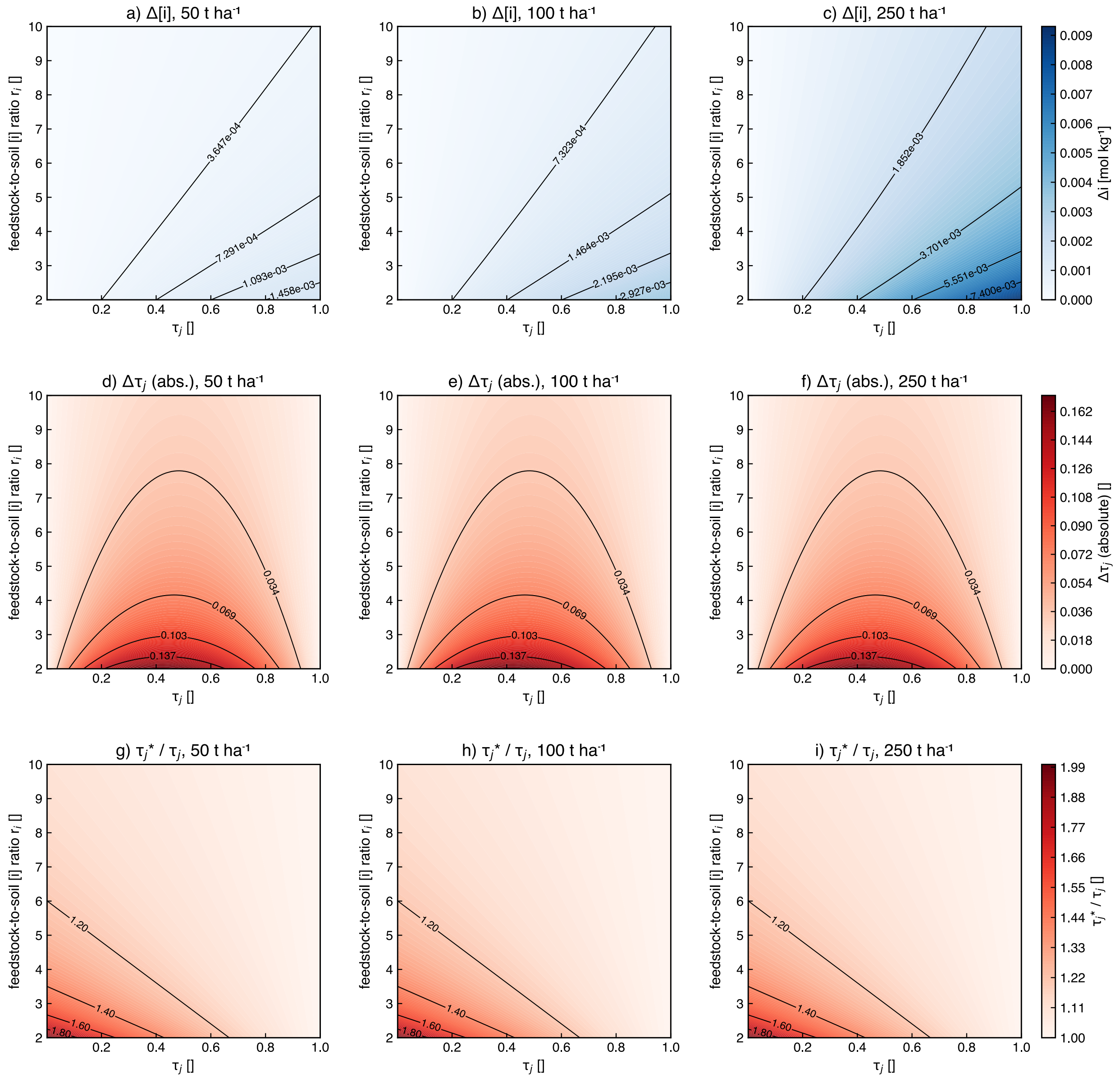

Figure S6: Immobile element enrichment and its effect on dissolution estimates for different application amounts (50, 100, and 250 t ha^{-1}; columns). The dissolution of added rock powder increases the immobile element concentration of topsoils of constant volume as the lost rock powder is replaced by soil from the bottom of the soil column (a-c). The difference between erroneously high τ_j when not taking this process into account and the actual τ_j is shown in d-f for absolute values (τ_j* - τ_j) and in g-i relative to the respective true τ_j.*

S3.1.2 Sensitivity of the simplified case to its assumptions being violated

The simplified SOMBA framework assumes that the density and mobile element concentration of the weathered feedstock endmember are equivalent to those of the baseline soil. We estimate the bias introduced when these assumptions are violated by systematically perturbing the weathered

feedstock density (ρ_{wf}) and mobile element concentration ($[j]_{wf}$) relative to soil values (ρ_s, $[j]_s$). The analysis was conducted across a two-dimensional parameter mesh ranging from 0.75–1.25 for both ρ_{wf}/ρ_s and $[j]_{wf}/[j]_s$. For each grid point, the true post-weathering composition was calculated using the full, non-simplified SOMBA mass-balance model (SOMBA_end), after which the same system was inverted with the simplified SOMBA formulation (SOMBA_tau_meta_simplified) to recover apparent dissolution fractions (τ) and application amounts (a).

Biases were quantified as the ratio between recovered and true values ($\tau_{detected}/\tau_{true}$ and $a_{deteced}/a_{true}$), allowing direct visualization of systematic deviations caused by departures from the simplifying assumptions. Simulations were performed for two true dissolution fractions (τ_{true} = 0.25 and 0.50) and three application rates (a = 50, 100, and 250 t ha^{-1}). The results are summarized in Figure S7 (τ_j) and Figure S8 (a), which show that deviations in ρ_{wf} and $[j]_{wf}$ primarily bias τ_j when the simplified framework is applied to systems that in reality follow the non-simplified three-endmember balance. Biases on both τ_j as well as a change with true τ_j values, with bias on detected τ_j decreasing with true τ_j and vice versa for a.

Overall, the analysis demonstrates that the simplified SOMBA framework remains robust within moderate deviations (±10–25%) in density and composition of the weathered feedstock.

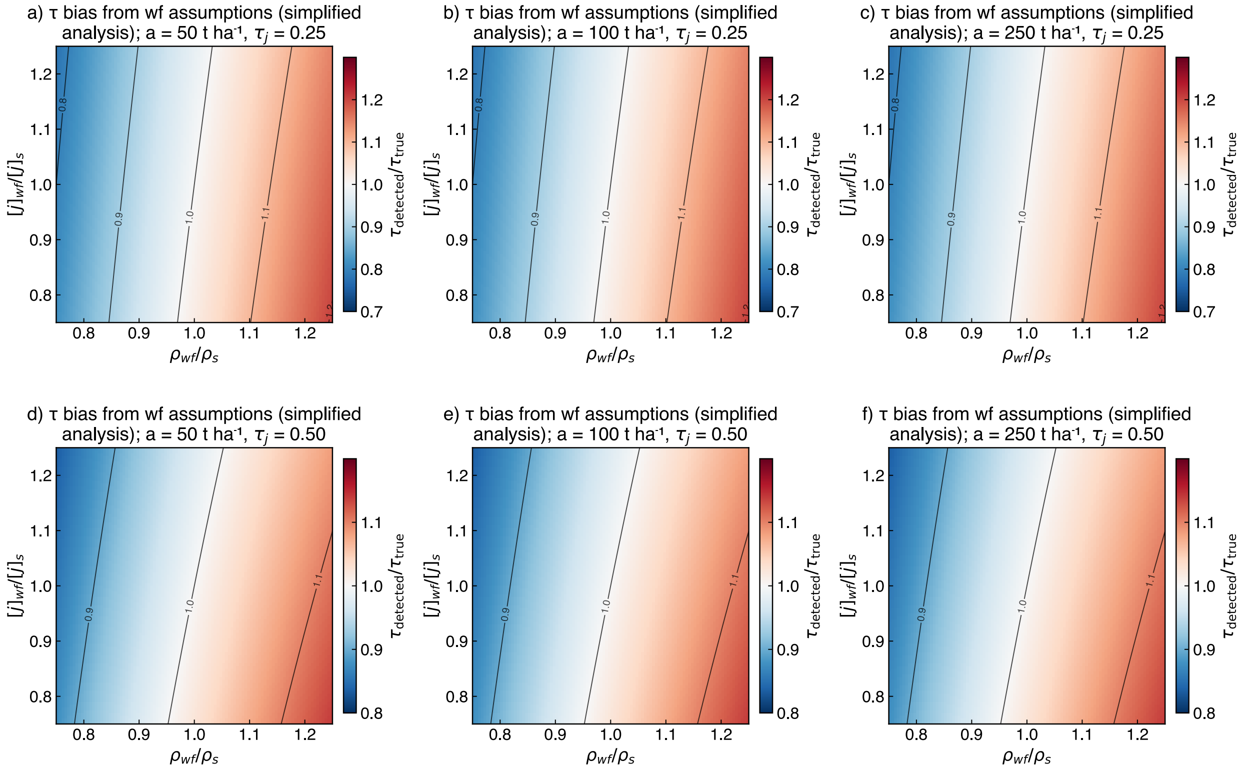

Figure S7: Sensitivity of the simplified SOMBA framework to violations of its underlying assumptions regarding the weathered-feedstock endmember. Each panel shows the bias in detected dissolution fractions (τ_j), expressed as $\tau_{detected}/\tau_{true}$, resulting from modeled deviations in weathered-feedstock density (ρ_{wf}) and mobile-element concentration ($[j]_{wf}$) relative to soil values (ρ_s, $[j]_s$). The parameter space (0.75–1.25 for both ratios) represents systematic violations of the simplified framework's assumptions that $\rho_{wf} = \rho_s$ and $[j]_{wf} = [j]_s$. Rows correspond to true dissolution fractions (τ_{true} = 0.25, 0.50) and columns to true application amounts (a_{true} = 50, 100, 250 t ha^{-1}).

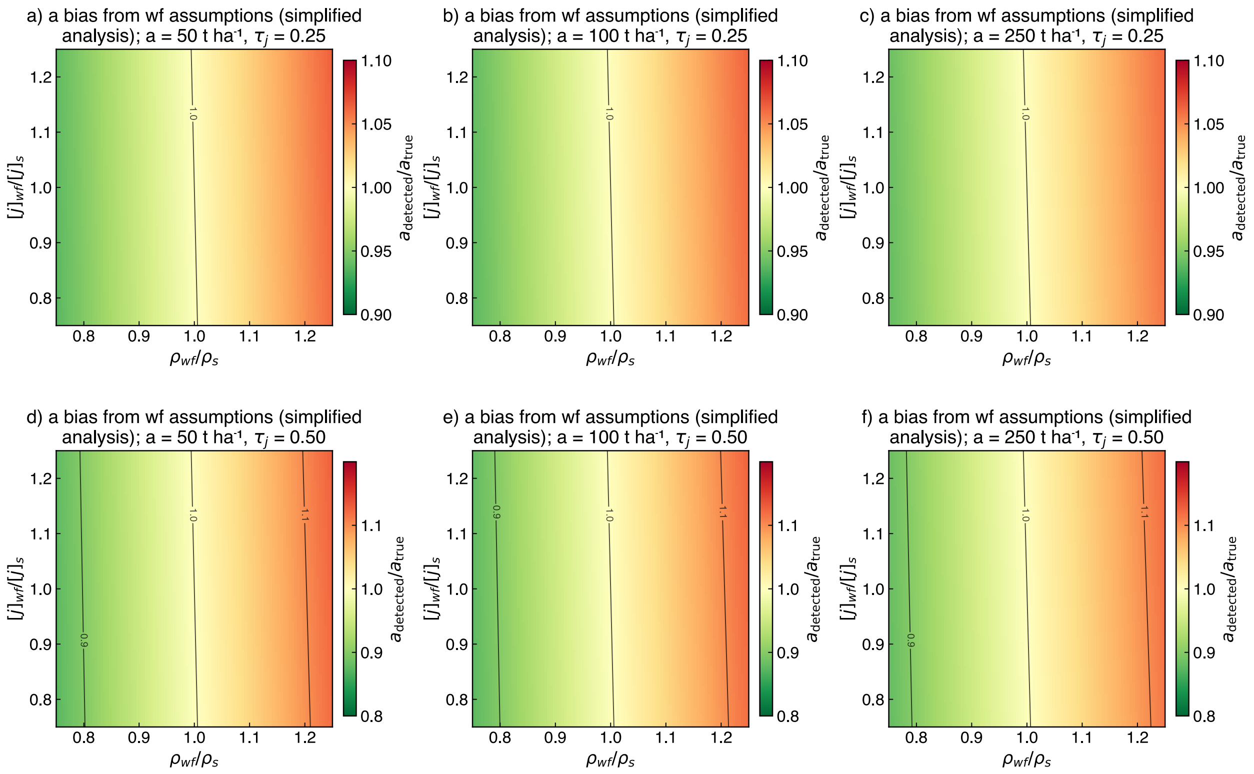

Figure S8: Sensitivity of the simplified SOMBA framework to violations of its underlying assumptions regarding the weathered-feedstock endmember. Each panel shows the bias in detected application amounts (a), expressed as $a_{detected}/a_{true}$, resulting from modeled deviations in weathered-feedstock density (ρ_{wf}) and mobile-element concentration ($[j]_{wf}$) relative to soil values (ρ_s, $[j]_s$). The parameter space (0.75–1.25 for both ratios) represents systematic violations of the simplified framework's assumptions that $\rho_{wf} = \rho_s$ and $[j]_{wf} = [j]_s$. Rows correspond to true dissolution fractions (τ_{true} = 0.25, 0.50) and columns to true application amounts (a_{true} = 50, 100, 250 t ha^{-1}).

S3.1.3 Sensitivity to mischaracterization of soil and feedstock density in the simplified framework

The simplified SOMBA framework relies on bulk density estimates of soil (ρ_s) and feedstock (ρ_f) to calculate the relative mass contributions of rock powder and soil within the sampled volume. In practice, these densities are commonly derived from field measurements or literature constraints and may not accurately reflect the true physical densities present in situ ($\rho_{s,true}$, $\rho_{f,true}$). To assess the impact of such discrepancies, we simulated systematic ±25% variations in the true soil and feedstock densities relative to their assumed values while holding all other parameters constant when estimating post-weathering composition (SOMBA_end_simplified). When these data are used to infer τ_j and a from the mass balance approach (SOMBA_tau_meta_simplified), we assume the erroneous nominal densities for the entire parameter space.

This analysis is not performed for calculated dissolution fractions (τ_j) as they are invariant to these density variations because the terms cancel in the underlying mass-balance relationships when quantifying τ_j. In contrast, the estimated application amount (a) is sensitive to density misestimation, as both soil and feedstock densities influence the volumetric scaling between true and assumed system states. Findings do however not depend on application amount and/or dissolution fraction (Figure S9). Overestimation of soil density (true-to-assumed ratio <1) leads to overestimation of applied rock powder amounts, while the opposite occurs when soil density is underestimated. Biases of up to ±30% can arise across the tested parameter space, depending on the direction and magnitude of the density mismatch.

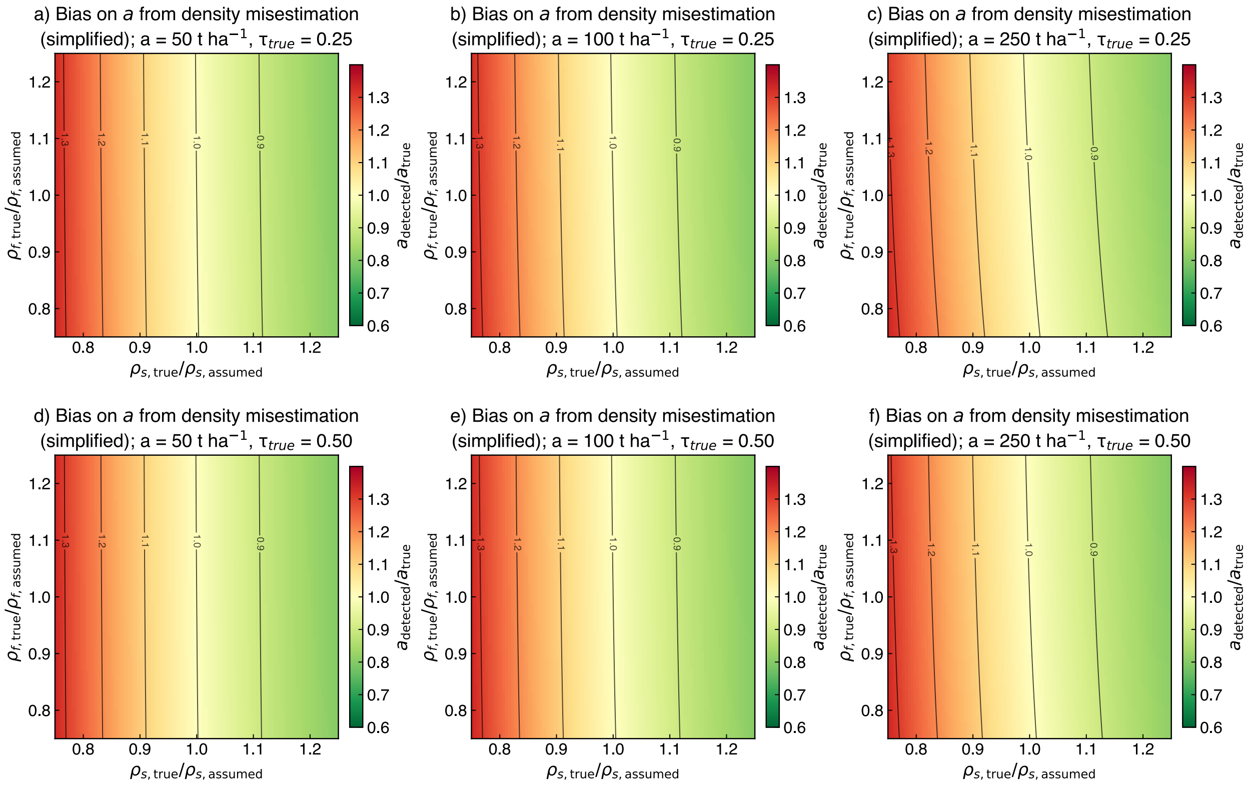

Figure S9: Bias in estimated rock powder application amounts (a) due to misestimation of soil (ρ_s) and feedstock (ρ_f) densities in the simplified SOMBA framework. Each panel shows the ratio $a_{detected}/a_{true}$ for combinations of true-to-assumed density ratios ($\rho_{s,true}/\rho_{s,assumed}$ and $\rho_{f,true}/\rho_{f,assumed}$). Rows correspond to true dissolution fractions (τ_{true} = 0.25, 0.50), and columns to application rates (a_{true} = 50, 100, and 250 t ha^{-1}). Results indicate that derived application amounts can be systematically over- or underestimated by up to approximately 30% within realistic density uncertainty ranges, irrespective of application amount or dissolution fraction.

S3.1.4 Sensitivity to feedstock and weathered-endmember density mis-quantification in the non-simplified framework

The non-simplified SOMBA framework explicitly incorporates the densities of both the fresh feedstock (ρ_f) and its weathered or partially dissolved residue (ρ_{wf})—and in contrast to the simplified approach these do not cancel out for the quantification of τ_j. To assess the sensitivity of the framework to such uncertainty, we systematically varied the true feedstock and weathered-feedstock densities ($\rho_{f,true}$, $\rho_{wf,true}$) by ±25% around their assumed values when estimating post-weathering composition (SOMBA_end), while the inversion (SOMBA_tau_meta) continued to assume constant densities (ρf, ρwf).

The resulting simulations quantify the extent to which mismatches between true and assumed densities bias the recovered dissolution fractions (τj; Figure S10) and application rates (a; Figure S11). Within the parameter space tested, both quantities exhibit systematic but distinct responses. For both τ_j and a, overestimating ρ_f (true-to-estimated ratio < 1) while underestimating ρ_{wf} causes systematic overestimation of both τ_j and a. For τ_j biases are larger at lower true τ_j values where they can be >30%. For a, biases increase with true τ_j, and are generally low at low dissolution fractions. Findings vary little with changing application amounts.

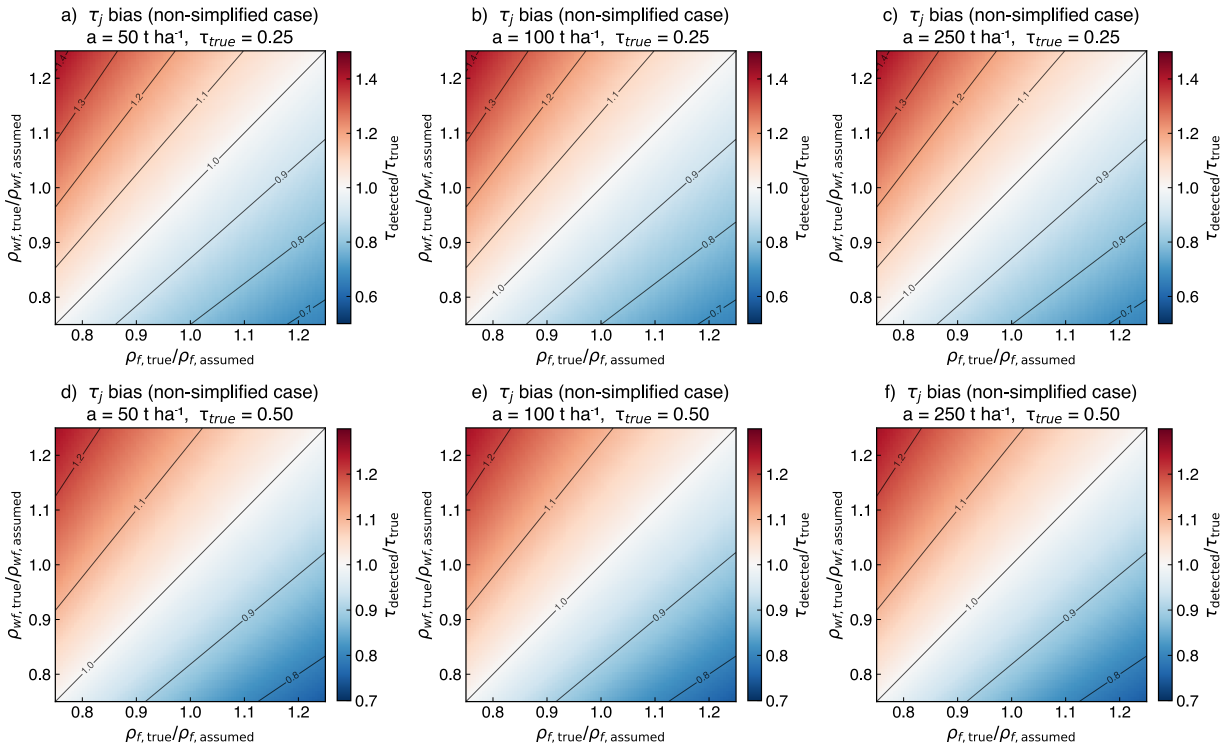

Figure S10: Bias in recovered dissolution fractions (τ_j) resulting from misestimation of feedstock (ρ_f) and weathered feedstock (ρ_{wf}) endmember densities in the non-simplified SOMBA framework. Each panel shows the ratio $\tau_{detected}/\tau_{true}$ for combinations of true-to-assumed density ratios ($\rho_{f,true}/\rho_{f,assumed}$ and $\rho_{wf,true}/\rho_{wf,assumed}$). Rows correspond to true dissolution fractions ($\tau_{j,true}$ = 0.25, 0.50), and columns to application rates (a = 50, 100, and 250 t ha^{-1}).

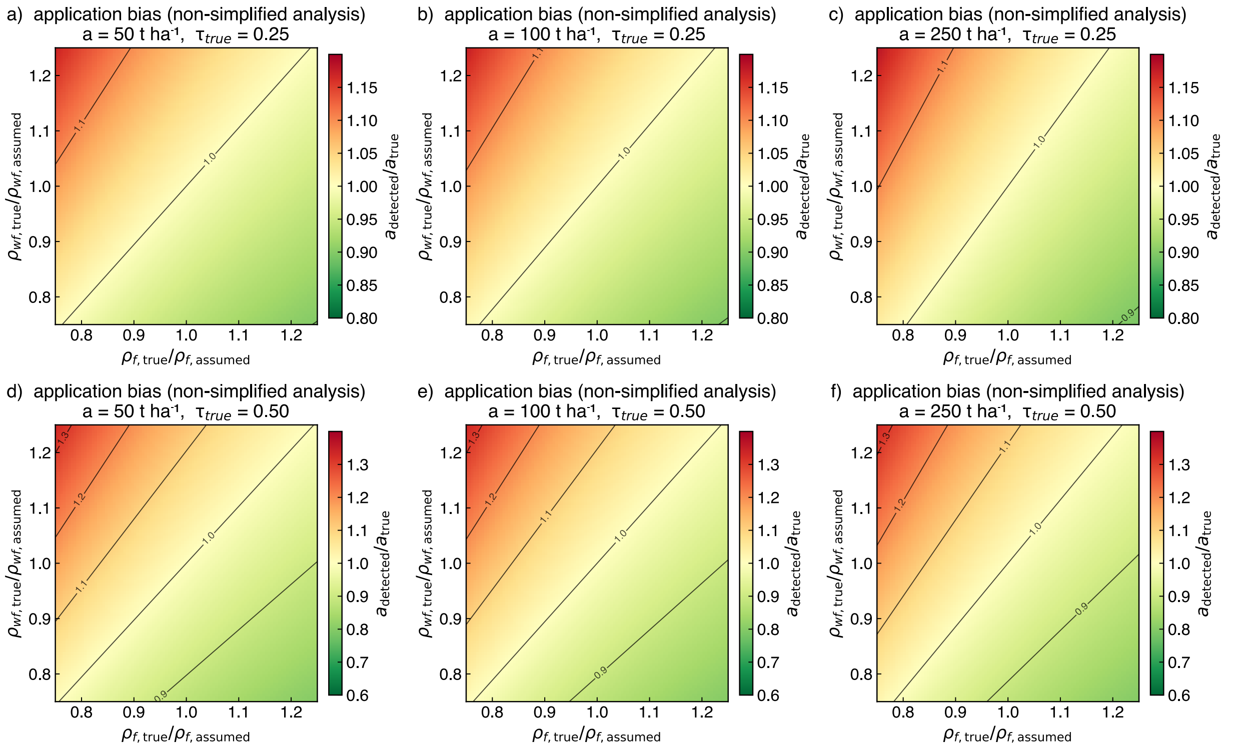

Figure S11: Bias in detected feedstock application amounts ($a_{detected}$) resulting from misestimation of feedstock (ρ_f) and weathered feedstock (ρ_{wf}) endmember densities in the non-simplified SOMBA framework. Each panel shows the ratio $a_{detected}/a_{true}$ for combinations of true-to-assumed density ratios ($\rho_{f,true}/\rho_{f,assumed}$ and $\rho_{wf,true}/\rho_{wf,assumed}$). Rows correspond to true dissolution fractions ($\tau_{j,true}$ = 0.25, 0.50), and columns to application rates (a = 50, 100, and 250 t ha^{-1}).

S3.4 Sensitivity to experimental depth errors in feedstock application amount detection

In practical field deployments of the SOMBA framework, discrepancies often arise between the *planned* and *actual* depths of feedstock incorporation (d_{mix}) and soil sampling (d_{sample}). Such deviations may result from variable soil resistance and non-ideal mixing equipment or inconsistent coring during post-application sampling. Because application amounts (a) are inferred from the mass balance within the sampled layer, mismatches between true and assumed layer boundaries alter the effective soil-to-feedstock ratio and can introduce systematic bias—even when elemental measurements are analytically precise. In contrast, dissolution fractions (τ_j) remain effectively invariant to these mismatches, as the same proportional depth error affects both the mobile and immobile elements equally.

Importantly, not all deviations between mixing and sampling depth should be interpreted as methodological failure. Different choices may be scientifically or logistically justified depending on the question being addressed. For instance, shallow sampling may intentionally focus on the most reactive near-surface zone, while deeper sampling may characterize redistribution of reaction products or assess soil buffering below the amended horizon. This analysis attempts to quantify how such deviations influence recovered application estimates when depth assumptions differ between the *true field state* (SOMBA_end_simplified) and the *model inversion* (SOMBA_tau_meta_simplified).

To reproduce this physical reality, we implemented a mechanistic correction for cases where the true sampling depth exceeds the true mixing depth ($d_{sample,true} > d_{mix,true}$). We compute the additional unmixed soil volume sampled below the mixed horizon ($V_{extra} = A \cdot (d_{sample,true} - d_{mix,true})$) and dilutes the mixed-layer composition accordingly. Elemental inventories (N) of both the mobile and immobile tracers are mass-weighted over the total sample mass ($m_{total} = m_{mixed} + m_{extra}$):

$$[j]_{sample} = \frac{N_{j,mixed} + N_{j,extra}}{m_{mixed} + m_{extra}} \quad \text{S81}$$

$$[i]_{sample} = \frac{N_{i,mixed} + N_{i,extra}}{m_{mixed} + m_{extra}} \quad \text{S82}$$

This adjustment mirrors the physical process during coring: if the sampling depth extends beyond the mixed layer, the upper feedstock-bearing material becomes diluted by pristine soil beneath. Conversely, if the actual mixing depth is shallower than assumed, the sampled layer includes proportionally more feedstock-rich material, leading to apparent overestimation of the application rate.

To quantify these effects, three sampling-depth scenarios were simulated:

1. *Partial-depth sampling* (target $d_{sample} = 0.5\ d_{mix}$), representing shallow coring relative to the intended mixed depth.
2. *Depth-equivalent sampling* (target $d_{sample} = d_{mix}$), representing mixing and sampling to equal depths.

3. *Depth-oversampling* (target $d_{sample} = 2\ d_{mix}$), where the sampling depth extends below the mixed layer.

Across all tested dissolution fractions (τ_j = 0.25, 0.50) and application rates (50, 100, and 250 t ha^{-1}), dissolution fraction estimates remained invariant, as both mobile and immobile elements are diluted in parallel. Application estimates, however, showed consistent and physically intuitive trends. In the case where sampling depth is much lower than mixing depth, if mixing depth is less than assumed, the application amount is overestimated because lower mixing depth means feedstock is more concentrated in the samples volume (**Error! Reference source not found.**g). In this case, inaccuracies in sampling depth have no impact—as sampling stays within the mixed layer the change in volume does not impact elemental concentrations. For cases where sampling depth is much deeper than mixing depth, inaccuracies in mixing depth do not impact detected application rates, but if sampling depth was lower than planned, application amounts are overestimated because the mixed layer makes up a larger proportion of the sampled layer and dilution by background soil is less important than assumed (**Error! Reference source not found.**i). Both of these processes operate in the case where planned sampling and mixing depth are equivalent (**Error! Reference source not found.**h), and the overall potential for overestimating application amounts is lowest.

S3.2 Impact of feedstock mass loss on base cation and immobile element concentrations

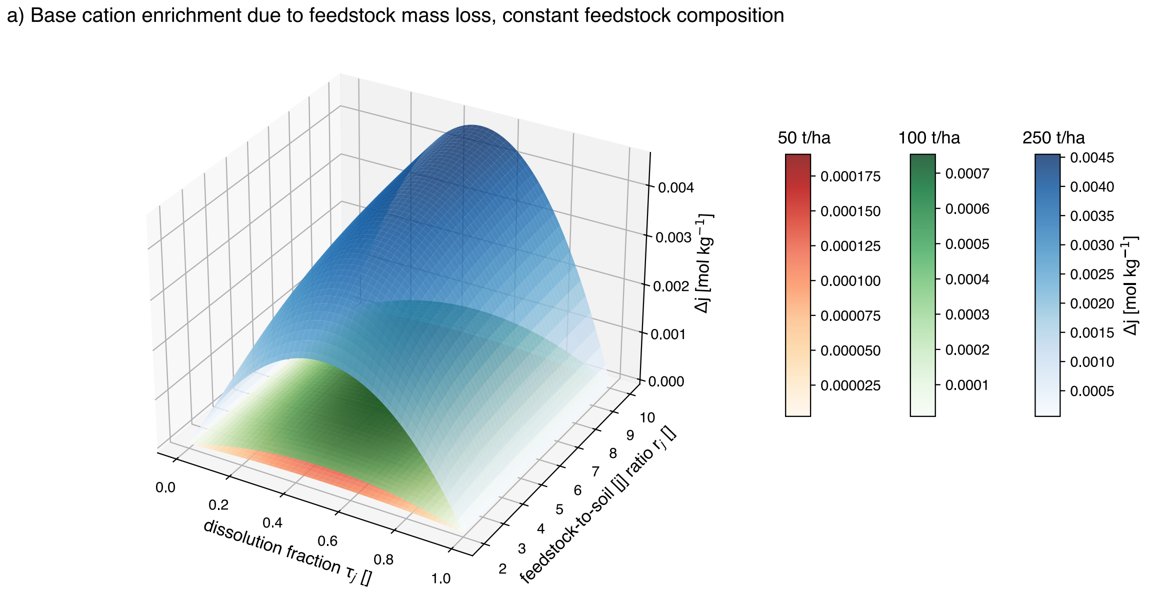

Figure S12 Change of base cation concentration as a result of feedstock mass/volume loss from the system considering constant feedstock compositions.

Calculated as:

$$\Delta j = [j]_{n=t} - [j]^*{}_{n=t} \quad \text{S83}$$

Where $[j]_{n=t}$ is equation from S72, and $[j]^*{}_{n=t}$ calculated from a linear loss of base cations relative to the dissolution fraction:

$$[j]^*{}_{n=t} = [j]_s + ([j]_{n=0} - [j]_s)(1 - \tau_j) \quad \text{S84}$$

Where $[j]_{n=0}$ is equation from S64.

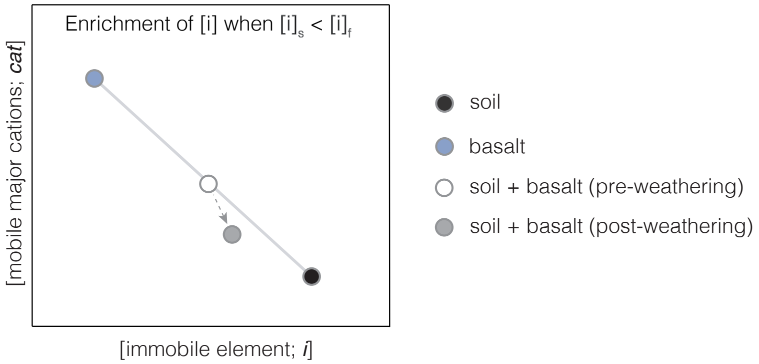

Figure S13: Sketch of the impact of enrichment of post-weathering soil immobile element concentrations when the feedstock immobile element concentration is lower than in soil.

S4 Impact of deployment parameters and soil composition on signal-to-noise analysis

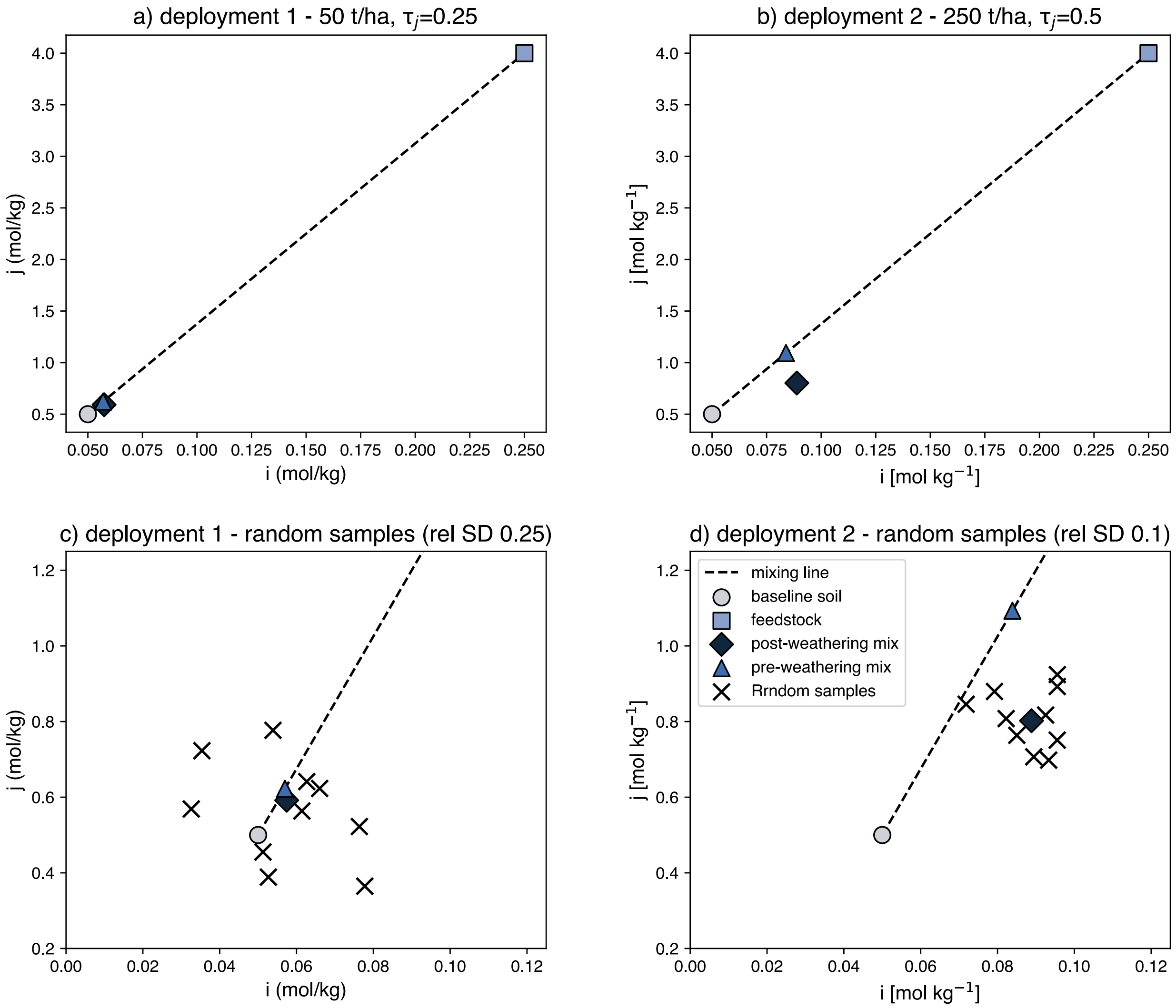

*Figure S14: Two exemplary EW deployments of 50 t ha^{-1}, τ_j of 0.25 and relative 1SD on sampled soil compositions of 25% (a and c) and 250 t ha^{-1}, τ_j of 0.5 and 1SD on soil samples of 10% (b and d), representative for a low- and high- resolvability scenarios. Panels c and d show the compositions of 10 random samples generated for the post-weathering soil-feedstock mixture (exact composition and associated sample τ_j values listed in **Error! Reference source not found.**).*

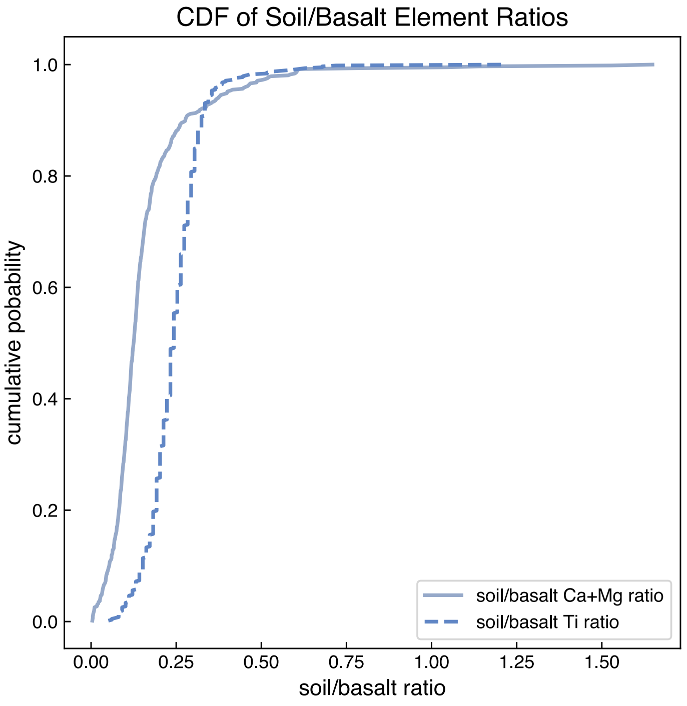

Figure S15: Cumulative distribution plot of the ratio of agricultural soil (LandCover2 = “Row Crops” and “Small Grains”) composition [1] to US-basalt composition [4]. As the ratio increases above the value of 0.2 defined here as a cut off for soils suitable for SOMBA, the fraction of soils fulfilling this condition quickly increases.

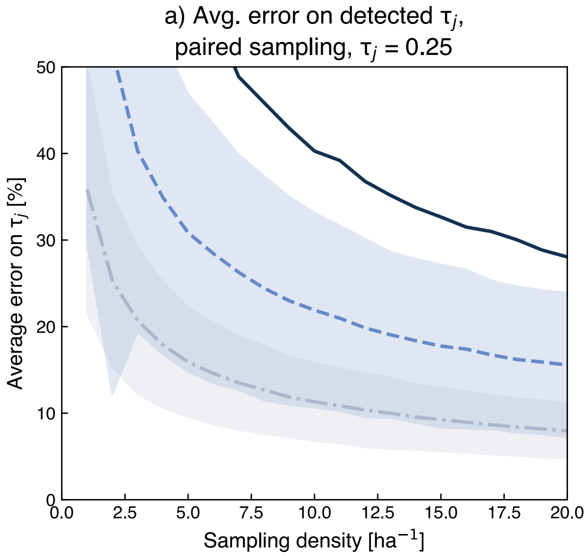

Figure S16: Average errors on detected dissolution fractions for two simulated mass transfer coefficients (τ_j = 0.25 in a, τ_j = 0.5 in b). The simulations are based on US soil [1] and basalt [4] compositions considering soils with base cation and Ti concentrations at least 2 times lower than basalt (in contrast to Figure 4, which shows the same for soil concentrations that are at least 5 times lower). The simulated in-field soil heterogeneity is based on the novel dataset presented in ***Error! Reference source not found.***.

References

(1) Smith, D. B.; Cannon, W. F.; Woodruff, L. G.; Solano, F.; Kilburn, J. E.; Fey, D. L. Geochemical and Mineralogical Data for Soils of the Conterminous United States. *U.S. Geological Survey Data Series* **2013**, *801* (April), 1–26. https://doi.org/10.3133/ds801.

(2) Potapov, P.; Turubanova, S.; Hansen, M. C.; Tyukavina, A.; Zalles, V.; Khan, A.; Song, X. P.; Pickens, A.; Shen, Q.; Cortez, J. Global Maps of Cropland Extent and Change Show Accelerated Cropland Expansion in the Twenty-First Century. *Nat Food* **2022**, *3* (1), 19–28. https://doi.org/10.1038/s43016-021-00429-z.

(3) Reershemius, T.; Kelland, M. E.; Davis, I. R.; D'Ascanio, R.; Kalderon-Asael, B.; Asael, D.; Suhrhoff, T. J.; Epihov, D. E.; Beerling, D. J.; Reinhard, C. T.; Planavsky, N. J. Initial Validation of a Soil-Based Mass-Balance Approach for Empirical Monitoring of Enhanced Rock Weathering Rates. *Environ Sci Technol* **2023**, *57* (48), 19497–19507. https://doi.org/10.1021/acs.est.3c03609.

(4) Lehnert, K.; Su, Y.; Langmuir, C. H.; Sarbas, B.; Nohl, U. A Global Geochemical Database Structure for Rocks. *Geochemistry, Geophysics, Geosystems* **2000**, *1* (5). https://doi.org/10.1029/1999gc000026.
